# Identification of ASF1A and HJURP by global H3–H4 histone chaperone analysis as a prognostic two-gene model in hepatocellular carcinoma

**DOI:** 10.1038/s41598-024-58368-1

**Published:** 2024-04-01

**Authors:** Yongkang Liu, Shihui Liu, Rui Jing, Congcong Li, Yongqi Guo, Zhiye Cai, Pei Xi, Penggao Dai, Lintao Jia, Hongli Zhu, Xiang Zhang

**Affiliations:** 1 National Engineering Research Center for Miniaturized Detection Systems, College of Life Science, Northwest University, Xi’an, 710069 Shaanxi China; 2 State Key Laboratory of Holistic Integrative Management of Gastrointestinal Cancers, Department of Biochemistry and Molecular Biology, Fourth Military Medical University, Xi’an, 710032 Shaanxi China; 3 The Ministry of Education Key Lab of Hazard Assessment and Control in Special Operational Environment, Fourth Military Medical University, Xi’an, 710032 Shaanxi China

**Keywords:** Cancer, Computational biology and bioinformatics, Biomarkers, Risk factors

## Abstract

Hepatocellular carcinoma (HCC) is a malignancy with poor prognosis. Abnormal expression of H3–H4 histone chaperones has been identified in many cancers and holds promise as a biomarker for diagnosis and prognosis. However, systemic analysis of H3–H4 histone chaperones in HCC is still lacking. Here, we investigated the expression of 19 known H3–H4 histone chaperones in HCC. Integrated analysis of multiple public databases indicated that these chaperones are highly expressed in HCC tumor tissues, which was further verified by immunohistochemistry (IHC) staining in offline samples. Additionally, survival analysis suggested that HCC patients with upregulated H3–H4 histone chaperones have poor prognosis. Using LASSO and Cox regression, we constructed a two-gene model (ASF1A, HJURP) that accurately predicts prognosis in ICGC-LIRI and GEO HCC data, which was further validated in HCC tissue microarrays with follow-up information. GSEA revealed that HCCs in the high-risk group were associated with enhanced cell cycle progression and DNA replication. Intriguingly, HCCs in the high-risk group exhibited increased immune infiltration and sensitivity to immune checkpoint therapy (ICT). In summary, H3–H4 histone chaperones play a critical role in HCC progression, and the two-gene (ASF1A, HJURP) risk model is effective for predicting survival outcomes and sensitivity to immunotherapy for HCC patients.

## Introduction

Liver cancer is the sixth most common cancer worldwide, with an estimated incidence of > 1 million cases by 2025, and the fourth leading cause of cancer-related death globally^1^. Hepatocellular carcinoma (HCC) is the most common form of liver cancer and accounts for ~ 90% of cases. Although clinical HCC treatments, including traditional therapy and immunotherapy, have improved significantly, unclear biomarkers for prognostic prediction and immunotherapy sensitivity impair the improvement of the survival rate of HCC patients^2^. Therefore, it is urgent to uncover effective molecular models to improve the survival rate.

Histone chaperones represent a class of regulators that have evolved to deposit and evict histones in a spatiotemporal manner throughout cell division, death and homeostasis. Their frequent misregulation in various cancers impacts tumor initiation and progression^3,4^. Recent studies concerning H3–H4 histone chaperones in several cancers illustrate their functions as tumor-promoting and/or useful biomarkers for clinical applications. Moreover, H3–H4 histone chaperones impact gene expression related to tumor initiation and progression by regulating DNA replication, DNA damage repair, and histone deposition^5^. Previous studies confirmed that some H3–H4 histone chaperones serve as useful biomarkers for prognosis evaluation and immunotherapy sensitivity prediction^6–9^. For example, the facilitates chromatin transcription complex (FACT), which consists of the histone chaperones SUPT16H and SSRP1, has been proven to be remarkably upregulated and contribute to tumor progression by promoting oxidative stress adaptation in HCC^10^. However, a systematic analysis of H3–H4 histone chaperones in HCC is still lacking. Therefore, we attempt to develop a prognostic model based on H3–H4 histone chaperones and assess its association with immune infiltration, which will be of great contribution for improving the prognosis of HCC patients.

In this study, we investigated the expression level and prognostic value of H3–H4 histone chaperones in HCC patients in The Cancer Genome Atlas (TCGA) and Gene Expression Omnibus (GEO) databases. LASSO and Cox regression were used to construct a prognostic two-gene (ASF1A and HJURP) model based on H3–H4 histone chaperones for HCC in TCGA-LIHC, which was evaluated in International Cancer Genome Consortium-Liver cancer-RIKEN (ICGC-LIRI) and GSE14520. Moreover, immunohistochemistry (IHC) of serial sections from HCC patients proved the elevated expression of ASF1A and HJURP in tumor tissues, and HCC patients in the high-risk group had poorer prognosis by IHC staining of HCC tissue microarrays. Intriguingly, HCC patients in the high-risk group had much more infiltration of immune cells, indicating a greater sensitivity to immune checkpoint therapy (ICT) for these patients. Taken together, the present study established an effective risk model for predicting survival outcome and sensitivity to immunotherapy in patients with HCC.

## Materials and methods

### Dataset sources and preprocessing

We obtained RNA-seq gene expression data and clinical records from The Cancer Genome Atlas (TCGA) database (https://portal.gdc.cancer.gov/) and the International Cancer Genome Consortium (ICGC) database (https://dcc.icgc.org/), respectively. The raw count matrix of RAN-seq was converted into transcripts per million (TPM), adding 1 to the value of TPM and using the logarithm of 2 as the base. The TPM matrix of RNA-seq was used for subsequent analysis. The GEPIA2 database (http://gepia2.cancer-pku.cn/#index) was used for H3–H4 histone chaperone expression analysis in pancancer. The ChEA3 database (https://maayanlab.cloud/chea3/) was used to predict the potential transcription factors of genes. The UniProt database (https://www.uniprot.org) was used to search for information about protein feature domains. Additionally, we downloaded the GSE121248, GSE33006, and GES14520 datasets from the Gene Expression Omnibus (GEO) repository. We integrated the two datasets (GSE121248 and GSE330006), comprising 113 samples from GEO, by removing the batch effect. This produced a larger combined cohort that provided a basis to validate the differential expression of H3–H4 histone chaperones between normal and tumor tissues. In total, we used 346 samples from the TCGA-LIHC cohort to construct a prognostic model, and 230 samples from the ICGC-LIRI cohort and 209 samples from the GSE14520 dataset were used to test the prognostic risk model.

### Patients and specimens

Serial sections from HCC patients were obtained from the Department of Pathology in Xijing Hospital. The clinicopathological characteristics of the HCC patients are listed in Supplementary Table S1. HCC tissue microarrays (TMAs) (D160Lv01S-ZK) were purchased from Xi’an Bioaitech Co., Ltd. (Xi’an China). The samples were analysed by IHC using anti-ASF1A and anti-HJURP antibodies according to the standard method and microarray instructions (the details of the primary antibodies for IHC are listed in Supplementary Table S2).

### Analysis of H3–H4 histone chaperone expression and patient survival

Wilcoxon tests were used to compare the expression of H3–H4 histone chaperones in normal and tumor tissues in HCC. Kaplan‒Meier (KM) survival curve analysis was implemented by the R software packages “survival” and “survminer”. High/low expression of H3–H4 histone chaperones was distinguished by the optimal cut-off value identified with the surv_cutpoint() function in the “survival” R package.

### Construction of the risk model

To construct the prognostic model, univariate Cox regression was used to identify whether the gene significantly correlated with survival outcome. Moreover, least absolute shrinkage and selection operator (LASSO) and multivariate Cox analyses were employed to further select reliable predictors. A forest plot was used to display the p value, HR and 95% CI of each variable through the “forestplot” R package. The risk score of each patient from the databases was assessed using RiskScore = Σ coefficient_mRNAn_ * expression level_mRNAn_. Then, KM survival curves were used to analyse the correlation of the risk score and prognosis of patients, and TimeROC analysis was performed to compare the prediction accuracy of the risk score in the ICGC-LIRI and GEO databases.

### Immunohistochemistry (IHC) staining and analysis

Tissue microarrays (TMAs) and sections were deparaffinized with xylene (3 × 15 min) and rehydrated with serial dilutions of ethanol (2 × 100%, 1 × 85%, and 1 × 75%, 5 min each) followed by rinsing in ddH2O. Heat-mediated antigen retrieval was performed by microwaving with EDTA pH 9.0. The sections were cooled on a decolorization shaker in PBS (3 × 5 min), immersed in 3% hydrogen peroxide, incubated at room temperature in darkness for 25 min, washed three times with PBS, and incubated for 30 min in blocking solution (3% BSA). The primary antibodies (the primary antibodies for IHC are listed in Supplementary Table S2) were diluted with PBS and incubated with sections for 1 h at 37 °C or overnight at 4 °C. The sections were washed three times for 5 min (3 × 5 min) with 1 × PBS on a shaker and then incubated with secondary antibody (HRP labelled) for 500 min at 37 °C. The sections were then washed 3 × 5 min with PBS and stained with the Immunohistochemical kit DAB chromogenic agent (Servicebio, G1211). The color development time was controlled under the microscope. The sections were counterstained with hematoxylin stain solution for approximately 3 min. Finally, tissues were dehydrated and mounted in Eukitt medium. Images were captured with a light microscope and analysed by using AIPATHWELL software (developed by Wuhan Servicebio Technology Co.).

### Differential expression analysis and functional annotation

A total of 346 samples with HCC were selected from the TCGA database and divided into high- and low-risk subgroups according to the median risk score. The Wilcoxon test was used to determine the differentially expressed genes (DEGs). The selection criteria for DEGs were as follows: |logFC|> 2 and *p* value < 0.05. GO and KEGG pathway enrichment analyses were performed with the “cluster-Profiler” R package^11–14^. Gene Set Enrichment Analysis (GSEA) was performed to investigate the functions correlated with different-risk subgroups of HCC by using the “clusterProfiler” R package. The gene sets used in GSEA were “c2.cp.kegg.v2022.1.Hs.entrez.gmt”, which was obtained from the Molecular Signature Database (MSigDB, https://www.gsea-msigdb.org/).

### Evaluation of immune cell infiltration

The CIBERSORT algorithm was used to quantify cell composition from gene expression profiles. In this study, the proportions of 22 immune cells between the high- and low-risk groups were analysed using CIBERSORT, running with 1000 permutations. To explore the correlation of the risk score with immune infiltration, we selected the samples from the top 50 and bottom 50 risk scores and performed single-sample GSEA (ssGSEA) using the R package “GSVA”. Spearman’s correlation analysis was used to evaluate the correlation between the abundance of immune cells and the risk score.

### Cell culture

The human HCC cell lines Hep3B, Huh-7, LM3, SNU-368, and SNU-739 and the human normal liver cell line LO2 were purchased from the National Infrastructure of Cell Line Resource and maintained in Dulbecco’s modified Eagle’s medium (Gibco, USA) or RPMI1640 (Gibco, USA) supplemented with 10% fetal bovine serum (Gibco, USA) at 37 °C with 5% CO_2_.

### Quantitative real-time polymerase chain reaction (qRT‒PCR)

RNA from cells was extracted using TRIzol reagent (Invitrogen, USA) according to the manufacturer’s instructions. cDNA was prepared from 1 μg RNA using the PrimeScript RT Reagent Kit Perfect Real Time Kit (Takara, Japan). qRT‒PCR was performed on a Bio-Rad CFX96 (Bio-Rad, USA) by using TB Green Premix Ex Taq II (Takara, Japan) according to the manufacturer’s protocol. The expression levels of the target genes were determined by amplification with specific primers with GAPDH as the internal control. All primers were obtained from Tsingke (Beijing, China), and the reactions were repeated three times. The primer sequences are listed in Supplementary Table S3.

### Western blotting

Cell extracts were prepared and lysed with RIPA buffer. The protein concentration was determined using a BCA kit. Samples were separated on 10% SDS‒PAGE gels and blotted onto nitrocellulose membranes (Millipore, USA). Membranes were incubated at 4 °C overnight with primary antibodies at the following concentrations: anti-ASF1A (1:000, 10784-1-AP, Proteintech), anti-HJURP (1:1000, 15283-1-AP, Proteintech), and anti-β-actin (1:2000, Sigma). The membranes were then washed three times with TBST and incubated with HRP-conjugated anti-rabbit IgG (1:20,000, 7074, Cell Signaling Technology) or anti-mouse IgG (1:20,000, 7076, Cell Signaling Technology) diluted in TBST at room temperature for 1 h. After a final wash with TBST, the membranes were developed with ECL reagents and visualized using a Tanon 5500 imaging system. The ratio of the expression of the indicated molecule to that of β-actin was determined using ImageJ software (National Institutes of Health, Bethesda, MD, USA).

### Statistical analysis

In this study, data analysis and visualization were performed using R software (version 4.0.2) and GraphPad Prism v9.0 software (GraphPad, La Jolla, CA, USA). Student’s t test, one-way ANOVA, Kruskal‒Wallis test, and Mann‒Whitney test were selected for statistical analysis of data according to the results of the test for normal distribution and test for homogeneity of variances. The statistical methods used for each result are described in the corresponding figure legends. Median survival time was identified by KM survival analysis, and survival was compared among groups with the log-rank test. Spearman’s correlation coefficients were calculated to determine the correlation between two continuous variables. All statistical tests were two-sided, and a *p* value < 0.05 was considered statistically significant.

### Ethics approval and consent to participate

The study involving human samples was approved by the Medical Ethics Committee of the First Affiliated Hospital of the Fourth Military Medical University (Approval Number: XJYYLL-2015625). The study was conducted in accordance with the local legislation and institutional requirements. Informed consent from all patients was obtained for participation in the study.

## Results

### Elevated expression of H3–H4 histone chaperones in hepatocellular carcinoma

**Figure 1:**
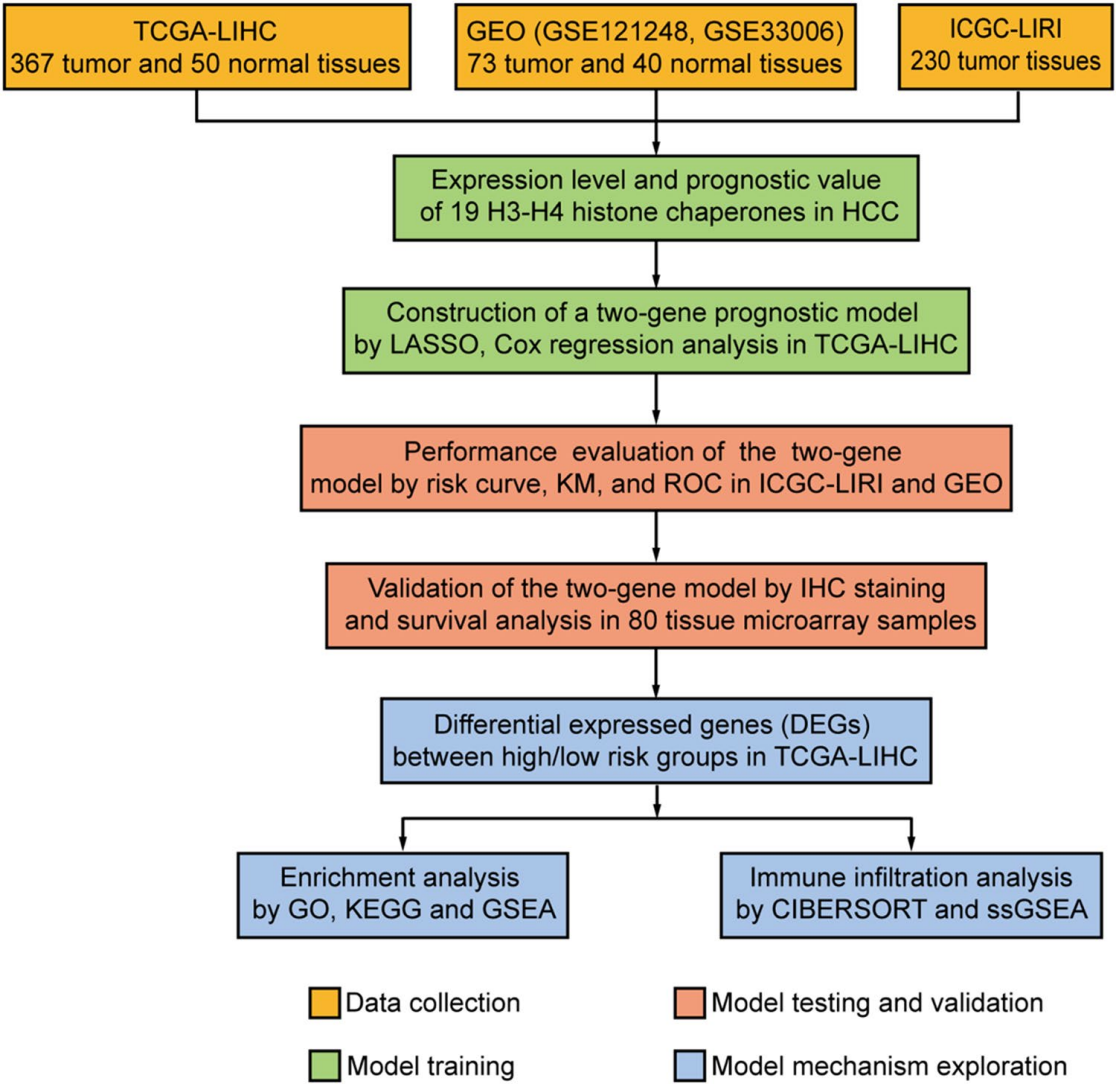
Flowchart of this study. First, the indicated datasets with prognostic information were collected from public databases. Second, the expression and prognostic value of all 19 H3–H4 histone chaperones in HCC were compared in the GEO and TCGA-LIHC databases. Third, a two-gene prognostic model based on H3–H4 histone chaperones was constructed by using LASSO and Cox regression analysis, and the accuracy and reliability of the prognostic model were evaluated in the ICGC-LIRI and GEO databases. Fourth, immunohistochemistry of serial sections of HCC patients was used to verify the difference between the expression levels of ASF1A and HJURP in tumor and normal tissues, and HCC tissue microarrays were used to validate the prognostic model. Finally, the signaling pathways involved in the prognostic model were analysed by GO, KEGG and GSEA analyses. CIBERSORT and ssGSEA were applied to explore the relationship between the risk score and immune cell infiltration.

**Figure 2:**
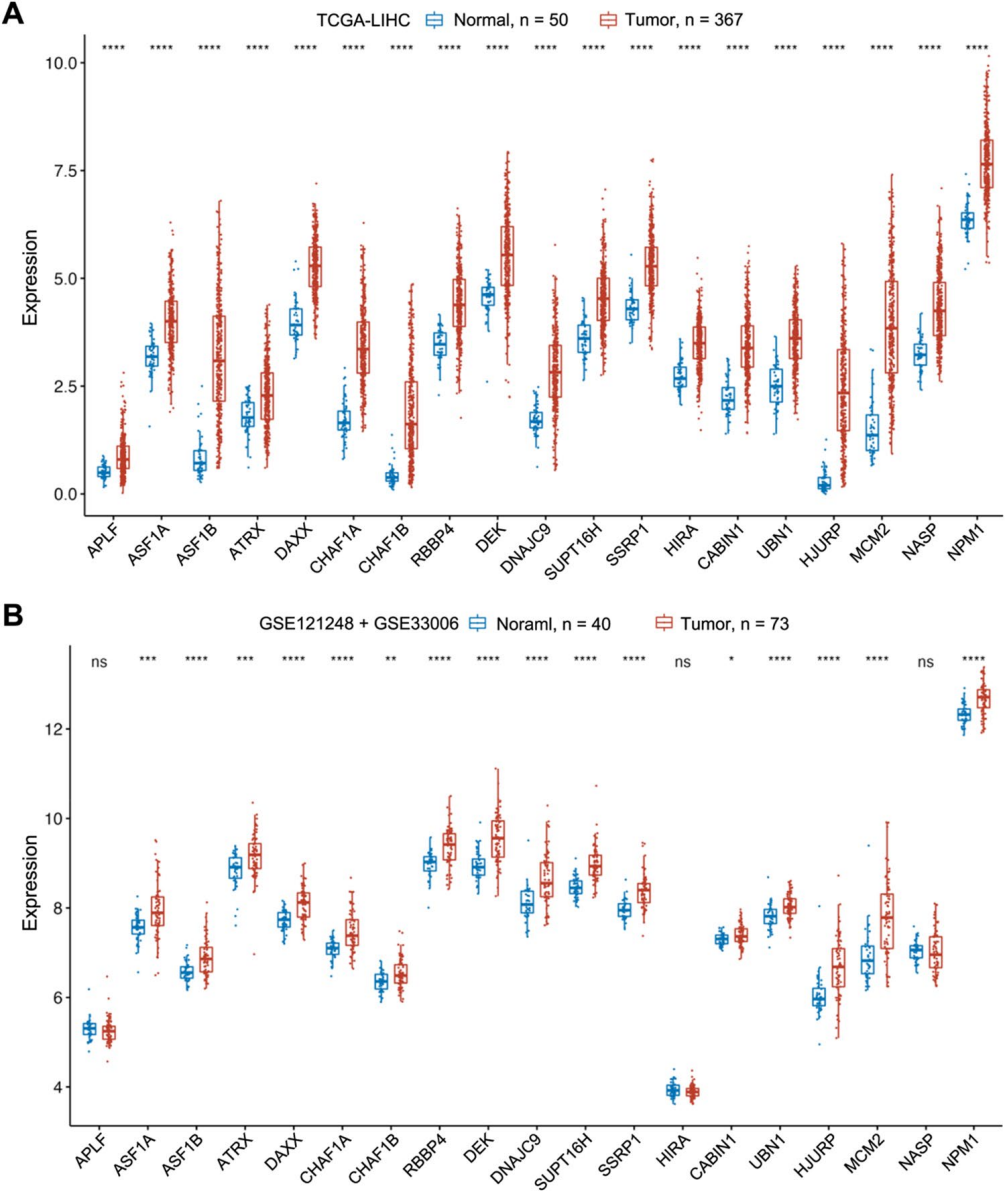
The elevated expression of H3–H4 histone chaperones in HCC. (**A**) The differences in 19 H3–H4 histone chaperones expressed in normal (n = 50) and tumor tissues (n = 367) in TCGA-LIHC. (**B**) The difference in 19 H3–H4 histone chaperones expressed in normal (n = 40) and tumor tissues (n = 73) in GEO (GSE121248 + GSE33006). Abbreviations: TCGA-LIHC, The Cancer Genome Atlas-Liver Hepatocellular Carcinoma; GEO, Gene Expression Omnibus; HCC, hepatocellular carcinoma; Kruskal‒Wallis test was performed to determine significance in (**A**,**B**); ns, not significant; *p < 0.05, **p < 0.01, ***p < 0.001, ****p < 0.0001.

A flowchart of this study is displayed in Fig. 1. We summarized all the known H3–H4 histone chaperones and their involved biological processes from publications (Table 1). We compared the expression of H3–H4 histone chaperones in normal and liver hepatocellular carcinoma (LIHC) tissues in TCGA, and we identified that all 19 H3–H4 histone chaperones are highly expressed in tumor tissues (Fig. 2A). A similar result was obtained by analysing the GSE121248 and GSE33006 datasets from the GEO database, and nearly all H3–H4 histone chaperones were significantly overexpressed in tumor tissues, except for APLF, HIRA and NASP (Fig. 2B). Overall, our comprehensive analysis showed that the expression of H3–H4 histone chaperones is significantly higher in HCC tissues than in normal tissues in the liver and suggested that H3–H4 histone chaperones are good candidate prognostic predictors.

**Table 1:** H3–H4 Histone chaperones in human species.

H3–H4 histone chaperone	Histone variant selectivity	Mediated biological process	References
APLF	H2A–H2B H3–H4	DNA repair, chromatin assembly	^34^
ASF1A	H3.3–H4	Proper folding of monomeric H3 and H4, expressed throughout the cell cycle; interacts with the HIRA histone chaperone in the nucleus	^4^
ASF1B	H3.1/2–H4	Expressed in the S-phase of the cell cycle, interacts with the CAF-1 complex on chromatin	^4^
ATRX-DAXX complex	H3.3–H4	H3.3 enrichment in heterochromatin region of telomeres with over repetitive DNA	^4,6,35^
CAF-1 complex (CHAF1A, CHAF1B, RBBP4)	H3.1/2–H4	DNA replication, new H3.1, H3.2 deposition	^7^
DEK	H3.3–H4	Chromatin organization	
DNAJC9	H3–H4	Recruiting of heat shock factors and release of misfolding H3–H4	^8^
FACT complex (SUPT16H, SSRP1)	H2A–H2B H3–H4	Histone eviction and recycling, transcriptional regulation; DNA repair and cell cycle	^9^
HIRA complex (HIRA, CABIN1, UBN1)	H3.3–H4	DNA repair and replication, new H3.3 deposition in transcriptionally active region	^36^
HJURP	CENP-A-H4	Chromatin deposition of CENP-A-H4	^4^
MCM2	H3–H4	Initiating nucleosome disassembly with FACT	^4^
NASP	H3–H4 H1	Assembling and folding of H3–H4 heterodimers	^4^
NPM1	CENP-A-H4	Chromatin deposition of CENP-A-H4	^4^

*APLF* aprataxin and PNKP like factor, *ASF1* antisilencing factor 1, *ATRX* α-thalassemia, mental retardation, X-linked syndrome, *DAXX* death domain-associated protein 6, *CAF-1* chromatin assembly factor 1, *RBBP4* retinoblastoma binding protein 4, *DEK* DEK proto-oncogene, *DNAJC9* DanJ heat shock protein family member C9, *FACT* facilitates chromatin transcription, *SUPT16H* suppressor of Ty 16 Homolog, *SSRP1* structure specific recognition protein 1, *HIRA* histone regulator A, *CABIN1* calcineurin binding protein 1, *UBN1* ubinuclein 1, *HJURP* Holliday junctions-recognition protein, *MCM2* minichromosome maintenance 2, *NASP* nuclear autoantigenic sperm protein, *NPM* nucleophosin.

Additionally, we analysed all tumor types containing normal samples in the TCGA database and found that different H3–H4 histone chaperones are expressed differently in different tumors. Of all the H3–H4 histone chaperones, HJURP is significantly highly expressed in almost all tumor types. In tumor types, almost all histone chaperones were significantly highly expressed in liver hepatocellular carcinoma (LIHC) and cholangiocarcinoma (CHOL) (Supplementary Fig. S1).

To explore whether elevated H3–H4 histone chaperones are regulated by common transcription factors in HCC. We predicted the potential transcription factors of these H3–H4 histone chaperones by using the ChEA3 database. There is no certain transcription factor that can regulate all histone chaperones. Instead, 23 transcription factors regulating more than 10 H3–H4 histone chaperones (Supplementary Table S4) were selected to analyse their expression changes with the progression of tumor stages in TCGA-LIHC. As displayed in Supplementary Fig. S2, five transcription factors (CREB1, CTCF, YY1, E2F1, MYBL2) were significantly elevated with tumor progression in HCC, indicating that these potential transcription factors may be responsible for the upregulation of H3–H4 histone chaperones in HCC.

Moreover, we investigated the expression of histone H3, H4 and CENP-A when their corresponding chaperones were upregulated in HCC. Because there are many genes encoding H3–H4 histone proteins and the process of assembling histone proteins into nucleosomes requires the participation of multiple chaperone molecules, the chaperone corresponding to histone proteins is not invariable and unique. Therefore, we used TCGA-LIHC data to determine the genes encoding histone H3 and H4 that are mainly expressed in liver cancer. We recognized that the genes encoding histones H3 and H4 are H3F3B and HIST1H4I in HCC, respectively (Supplementary Fig. S3A,B). We then conducted correlation analysis of H3F3B, HIST1H4I and CEPN-A with each H3–H4 histone chaperone (Supplementary Table S5, Supplementary Fig. S3C). H3F3B and CENP-A are significantly positively correlated with all H3–H4 histone chaperones, and HIST1H4I is positively correlated with most (11 out of 19) H3–H4 histone chaperones (*p* < 0.05), suggesting that the expression of histone levels may also be upregulated in HCC when their corresponding chaperones are upregulated.

### Construction of the prognostic model based on H3–H4 histone chaperones in HCC

First, for each H3–H4 histone chaperone, we analysed the overall survival probability of HCC patients with different expression levels. In this section, HCC patients were divided into a high-expression group and a low-expression group according to the optimal cut-off value. We observed significantly shorter overall survival (OS) in HCC patients with higher expression of most H3–H4 histone chaperones (Supplementary Fig. S4). Then, we used univariate Cox regression to analyse the relationship between H3–H4 histone chaperone expression, clinical factors (such as sex, age, and stage) and OS in HCC patients. Univariate Cox analysis indicated that clinical stages and the expression of most H3–H4 histone chaperones are significantly correlated with OS in HCC (Table 2).

**Table 2:** Univariate Cox regression analysis of H3–H4 histone chaperones in TCGA-LIHC.

Variates	HR (95% CI for HR)	*p* value	Significance
Gender	1.24 (0.5–1.2)	0.286	ns
Age	1.01 (1–1)	0.167	ns
Stage			
II	1.39 (0.84–2.3)	0.196	ns
III	2.37 (1.5–3.7)	0.000123	***
IV	5.57 (1.7–18)	0.00426	**
APLF	1.03 (0.86–1.2)	0.752	ns
ASF1A	1.67 (1.3–2.1)	2.94E−05	****
ASF1B	1.25 (1.1–1.4)	0.000586	***
ATRX	1.1 (0.9–1.3)	0.357	ns
DAXX	1.53 (1.1–2)	0.0045	**
CHAF1A	1.36 (1.1–1.7)	0.00175	**
CHAF1B	1.24 (1.1–1.4)	0.000491	***
RBBP4	1.52 (1.2–1.9)	0.000516	***
DEK	1.23 (1–1.5)	0.0328	*
DNAJC9	1.27 (1.1–1.5)	0.0122	*
SUPT16H	1.46 (1.1–1.9)	0.00499	**
SSRP1	1.79 (1.4–2.3)	1.41E−05	****
HIRA	1.37 (1–1.8)	0.0259	*
CABIN1	1.23 (0.97–1.6)	0.0853	ns
UBN1	1.21 (0.93–1.6)	0.158	ns
HJURP	1.37 (1.2–1.6)	1.30E–06	****
MCM2	1.35 (1.2–1.6)	1.63E−05	****
NASP	1.6 (1.3–2)	3.41E–05	****
NPM1	1.69 (1.3–2.1)	1.10E−05	****

*HR* hazard ratio, *95% CI* 95% confidence interval, *ns* not significant, *TCGA-LIHC* The Cancer Genome Atlas Liver Hepatocellular Carcinoma.

*，*p* < 0.05；**，*p* < 0.01；***，*p* <0.001；****，*p* < 0.0001

**Figure 3:**
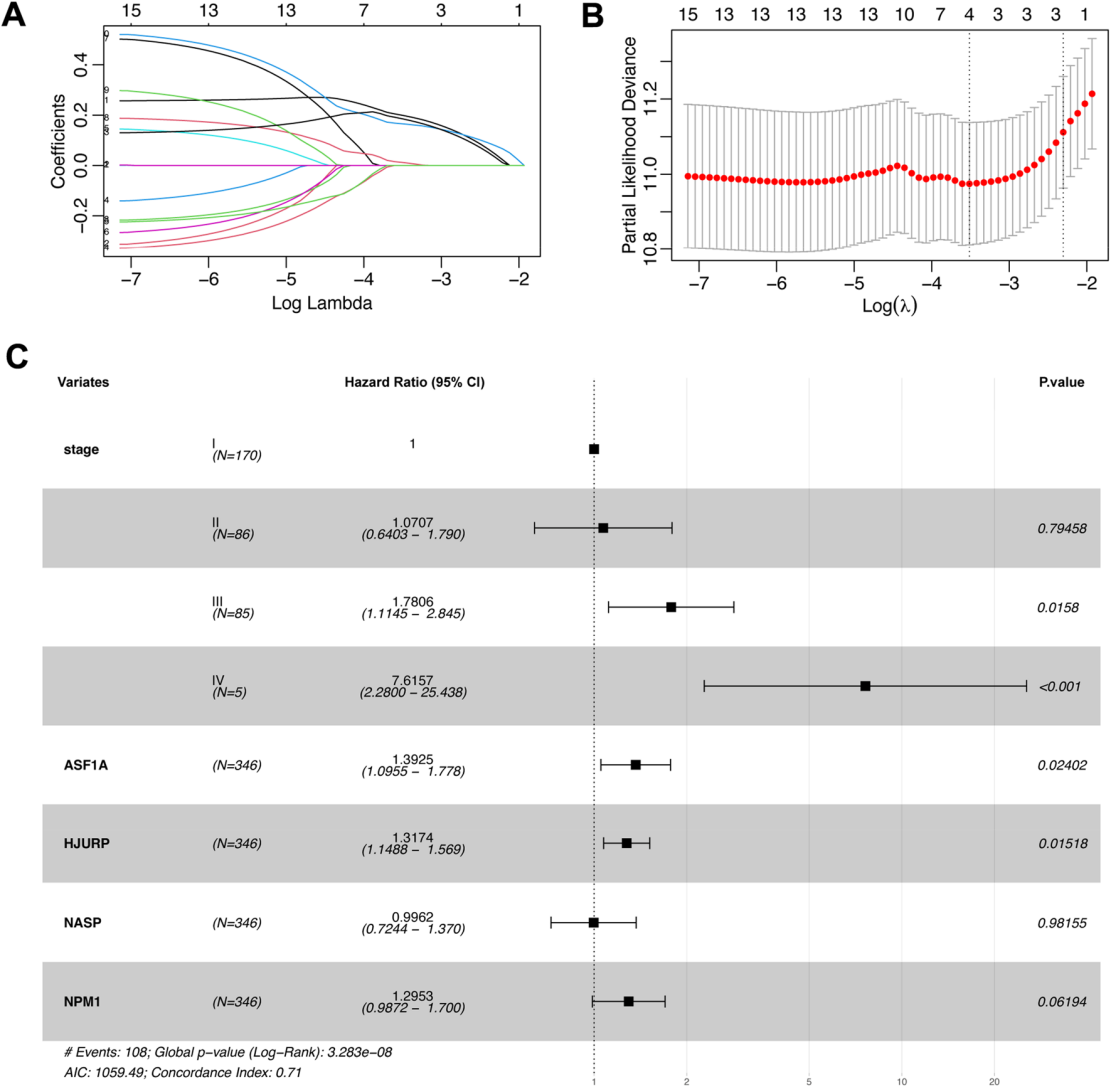
Construction of the risk model based on H3–H4 histone chaperones in HCC. (**A**) LASSO coefficient profile plots of each independent variable. (**B**) The partial likelihood deviance for the LASSO Cox regression analysis. (**C**) Multivariate Cox analysis of H3–H4 histone chaperones and clinical pathological variables. 95% CI, 95% confidence interval. *p* value < 0.05 was considered statistically significant.

Second, LASSO Cox regression analysis was used to reduce the number of candidate H3–H4 histone chaperones in the prognostic model. The change trajectory of each candidate H3–H4 histone chaperone is shown in Fig. 3A,B. After LASSO regression analysis, 4 histone chaperones (ASF1A, HJURP, NASP and NPM1) were subjected to multivariate Cox regression analysis to construct the final prognostic risk model (Fig. 3C, Supplementary Table S6).

Finally, two H3–H4 histone chaperones, ASF1A and HJURP, were identified to generate a risk model based on the univariate and multivariate Cox results. Furthermore, the analysis revealed that higher TNM stage and ASF1A or HJURP expression were independent prognostic factors for HCC (HR > 1, *p* < 0.05). The two-gene model formula was as follows: RiskScore = 0.343 * Expression_ASF1A_ + 0.247 * Expression_HJURP_.

### The two-gene model passes the prognostic prediction test in the ICGC-LIRI and GEO HCC cohorts

**Figure 4:**
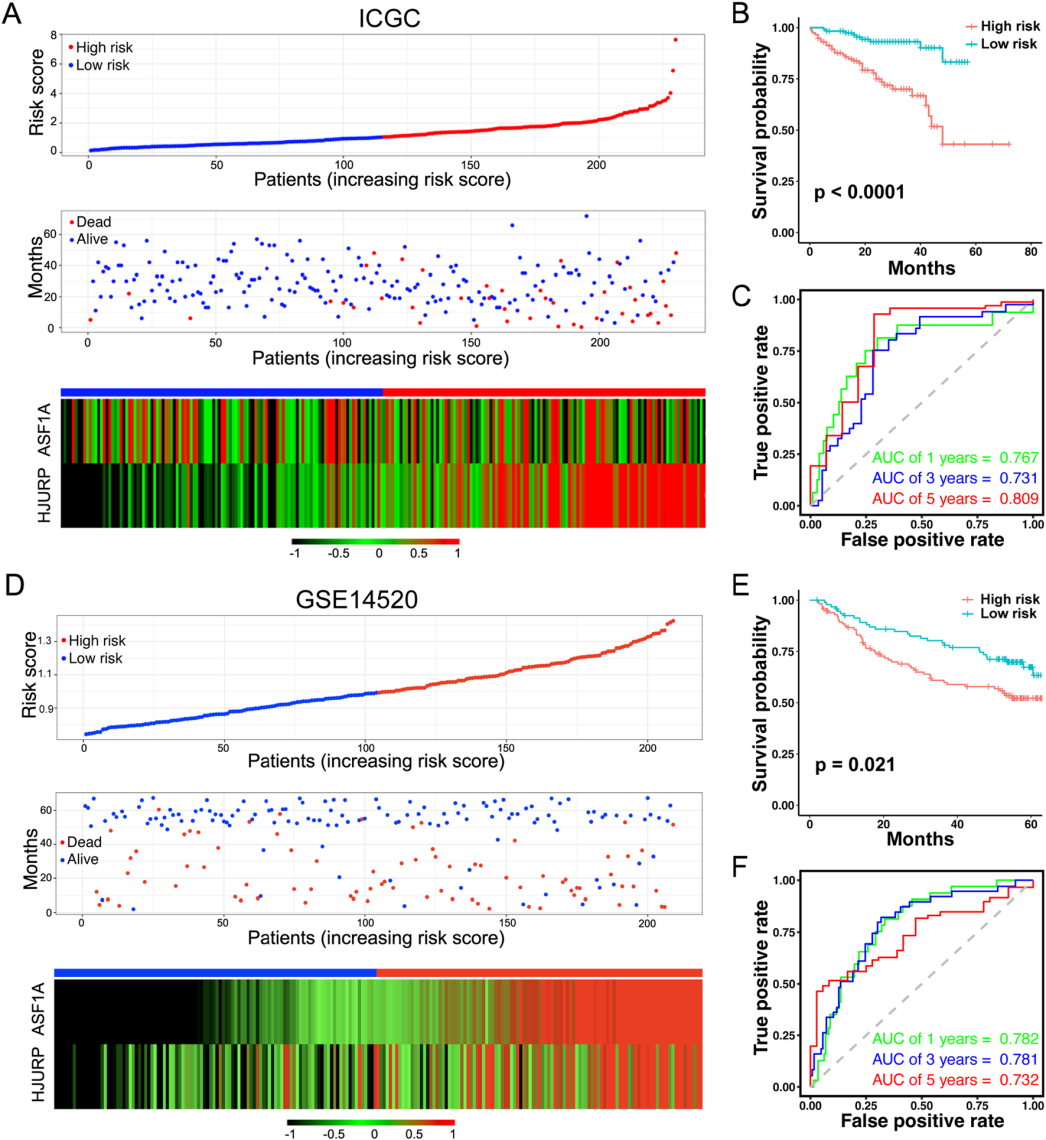
Evaluation of the predictive efficacy of the prognostic model in HCC. (**A**) The risk score distribution, patient status, ASF1A and HJURP expression heatmap for the ICGC-LIRI cohort. (**B**) Kaplan–Meier curve of the two-gene model for the ICGC-LIRI cohort. (**C**) Time-dependent receiver operating characteristic (ROC) curves of the risk model for the prediction of 1-, 3-, and 5-year survival in the ICGC-LIRI cohort. (**D**) The risk score distribution, patient status, ASF1A and HJURP expression heatmap for the GSE14520 cohort. (**E**) Kaplan–Meier curve of the two-gene model for the GSE14520 cohort. (**F**) ROC curves of the risk model for the prediction of 1-, 3-, and 5-year survival in the GSE14520 cohort.

After construction of the two-gene model, we first calculated the risk scores based on the model for each patient in the ICGC-LIRI cohort and plotted the risk score distribution of the patients. HCC patients were divided into high- and low-risk groups according to the median risk score. As expected, patients in the high-risk group had higher risks of death than those in the low-risk group (Fig. 4A). Survival analysis also exhibited poorer prognosis in patients with high-risk scores than in those with low-risk scores (Fig. 4B). The receiver operating characteristic (ROC) curve indicated that the risk score can perfectly predict the survival rate for HCC patients at 1, 3, and 5 years in the ICGC-LIRI cohort, and its areas of under the curves (AUCs) were 0.767, 0.731, and 0.809, respectively (Fig. 4C). To further evaluate this two-gene model, we tested it in another GEO dataset (GSE14520) (Fig. 4D). Similarly, the patients in the high-risk group presented a significantly shorter overall survival rate than those in the low-risk group (Fig. 4E). Moreover, the areas under the curves in the GSE14520 cohort were 0.782, 0.781, and 0.732 at 1, 3, and 5 years, respectively, indicating that the risk scores generated by this two-gene model can precisely predict the survival time of HCC patients (Fig. 4F). Additionally, we investigated whether a single gene from the two-gene model exhibited comparable prognostic performance to the two-gene model in HCC. ROC curves revealed that the AUC values for ASF1A or HJURP in predicting 1, 3, and 5-year survival rates among HCC patients were relatively low in ICGC and GSE14520 datasets (Supplementary Fig. S5). These findings suggest that ASF1A and HJURP are both essential for constructing the prognostic model. In summary, we created an excellent risk model for predicting the survival probability in HCC.

### Validation of the two-gene model in HCC clinical samples and cell lines

**Figure 5:**
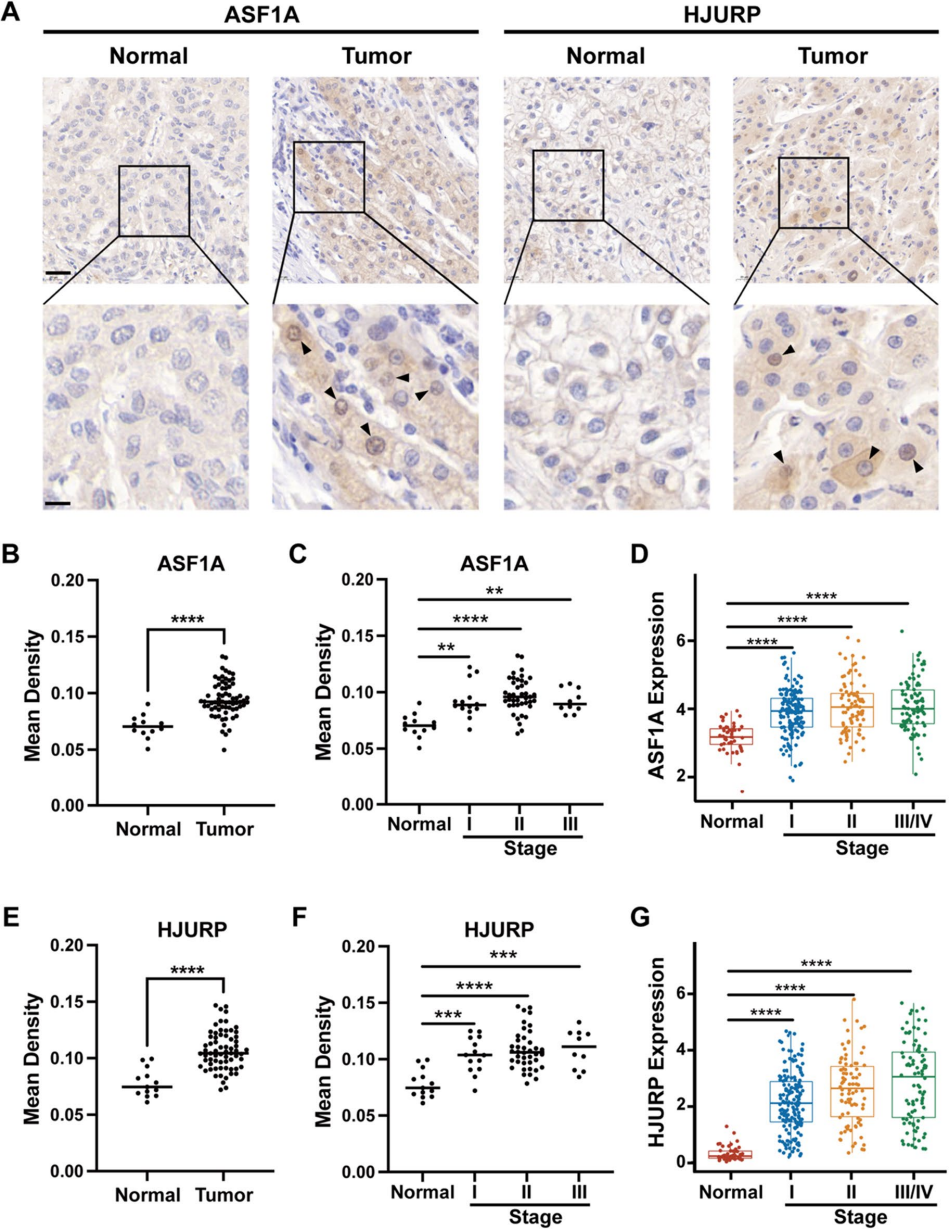
The expression levels of ASF1A and HJURP in serial sections of HCC and normal liver tissues. (**A**) Immunohistochemical (IHC) staining for ASF1A and HJURP in serial sections of HCC and normal liver tissues. Black arrows indicate cells positive for ASF1A or HJURP. Scale bar, top, 30 µm, bottom 10 µm. (**B**) Comparison of the mean density of ASF1A in HCC and normal liver tissues. (**C**) Comparison of the mean density of ASF1A in serial sections of HCC at different stages and normal liver tissues. (**D**) The boxplot of ASF1A expression in different stages of HCC and normal tissues in TCGA-LIHC. (**E**) Comparison of the mean density of HJURP in HCC and normal liver tissues. (**F**) Comparison of the mean density of HJURP in serial sections of HCC at different stages and normal liver tissues. (**G**) Boxplot of HJURP expression in different stages of HCC and normal tissues in TCGA-LIHC. Mean density, the average integrated optical density of positive pixel area analysis by AIPATHWELL software, and the value represents the relative level of the protein expressed in the tissue. Statistical significance was computed using Student's t test (**B**,**C**), ordinary one-way ANOVA (**D**,**E**), and the Kruskal‒Wallis test (**F**,**G**); **p < 0.01, ***p < 0.001, ****p < 0.0001.

To investigate the protein levels of ASF1A and HJURP in HCC and adjacent normal tissues, we first collected 62 clinical samples from Xijing Hospital. Immunohistochemical (IHC) staining showed that ASF1A and HJURP were expressed more highly in tumor tissues than in normal tissues (Fig. 5A,B,E). Meanwhile, IHC staining results also revealed that tissues from patients with a higher stage had higher levels of both ASF1A and HJURP (Fig. 5C,F). Intriguingly, the RNA levels of ASF1A and HJURP also gradually increased with tumor stage progression in the TCGA-LIHC data (Fig. 5D,G).

**Figure 6:**
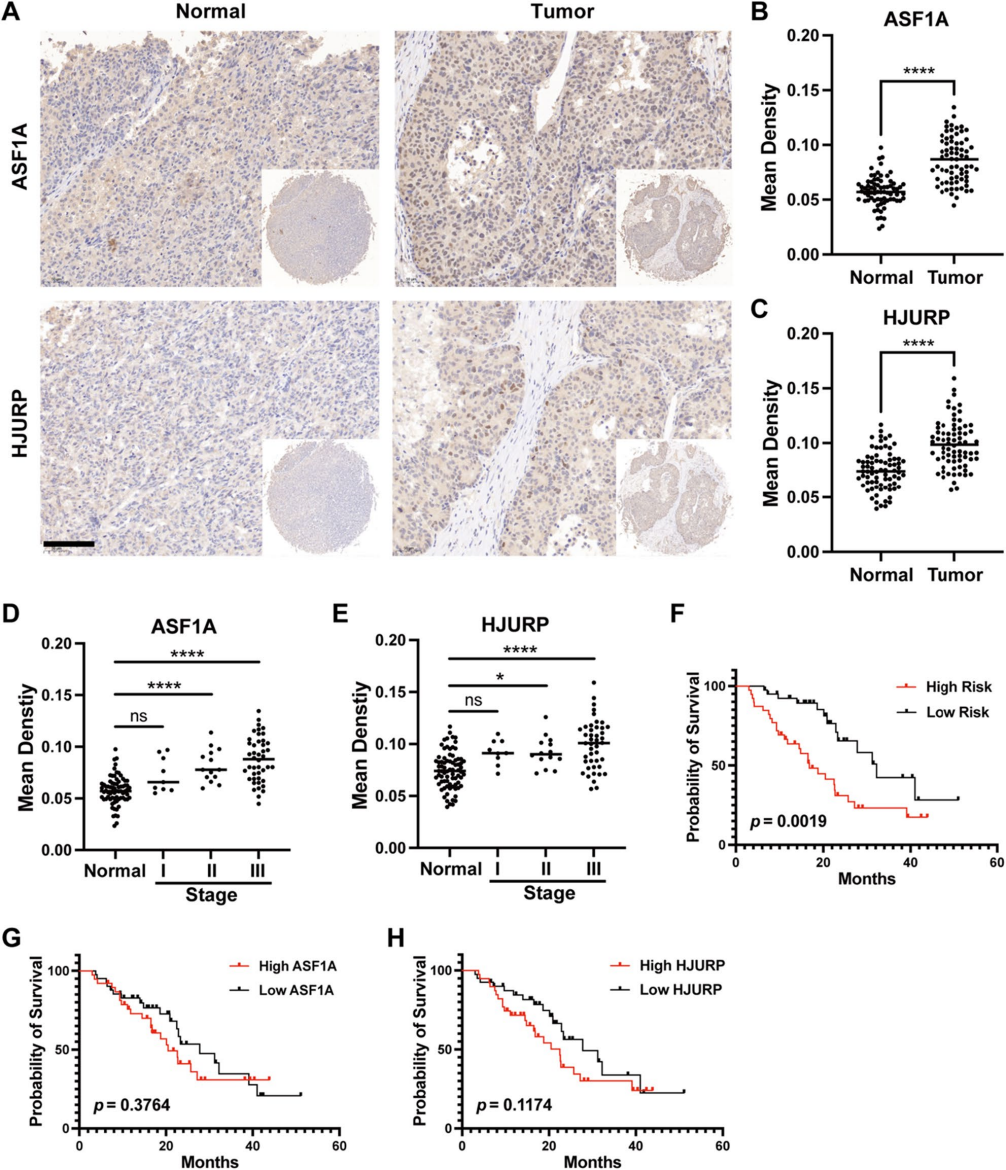
IHC and survival analysis of HCC tissue microarray. (**A**) IHC staining results of ASF1A and HJURP in HCC tissue microarrays. Scale bar, 100 µm. Comparison of the mean density of ASF1A (**B**) and HJURP (**C**) in HCC and normal tissue microarrays. Comparison of the mean density of ASF1A (**D**) and HJURP (**E**) in different stage HCC and normal tissue microarrays. (**F**) Kaplan–Meier curves of the expression of the two-gene model for the tissue microarray data. Kaplan–Meier curves of the expression of ASF1A (**G**) and HJURP (**H**) for the tissue microarray data. Mean density, the average integrated optical density of positive pixel area analysis by AIPATHWELL software, and the value represents the relative level of the protein expressed in the tissue. Statistical significance was computed using the Mann‒Whitney test (**B**), Student's t test (**C**), Kruskal‒Wallis test (**D**), and ordinary one-way ANOVA (**E**); ns, not significant; *p < 0.05, ****p < 0.0001.

To further validate the prognostic value of the two-gene model, we investigated ASF1A and HJURP levels in prognosis-containing HCC tissue microarrays (TMAs) (Supplementary Fig. S6). Similar to our previous observations in clinical samples, ASF1A and HJURP mainly localize in the nucleus and exist in the cytoplasm (Fig. 6A). Statistical analysis showed increasing mean densities of ASF1A and HJURP with tumor development and progression (Fig. 6B–E). Moreover, substituting the expression values of ASF1A and HJURP into the constructed two-gene model (RiskScore = 0.343 * Expression_ASF1A_ + 0.247 * Expression_HJURP_), the patients were divided into high-/low-risk groups based on the median risk score, and high-risk patients truly had a significantly worse prognosis (Fig. 6F), indicating that the two-gene model can accurately predict the prognosis of patients with HCC based on not only RNA levels but also proteins. Intriguingly, each gene between the two-gene model had no significance in predicting patient prognosis (Fig. 6G,H); therefore, ASF1A and HJURP are both necessary for prognostic analysis in HCC.

Moreover, further validation was performed in HCC cell lines. We used a normal hepatocyte-derived cell line (LO2) as the control and compared the relative expression levels of ASF1A and HJURP in LO2 and five hepatocellular carcinoma (HCC) cell lines (Hep3B, Huh-7, LM3, SNU-368, SNU-739) using qRT‒PCR) and WB experiments. As displayed in Supplementary Fig. S7, we confirmed that the expression of ASF1A and HJURP was significantly upregulated in most HCC cells compared to LO2 cells at both the RNA and protein levels. This conclusion is consistent with the results in Figs. 2, 5, and 6. Therefore, we confirmed that ASF1A and HJURP are elevated in HCC.

### Exploration of biological function underlying the two-gene model

**Figure 7:**
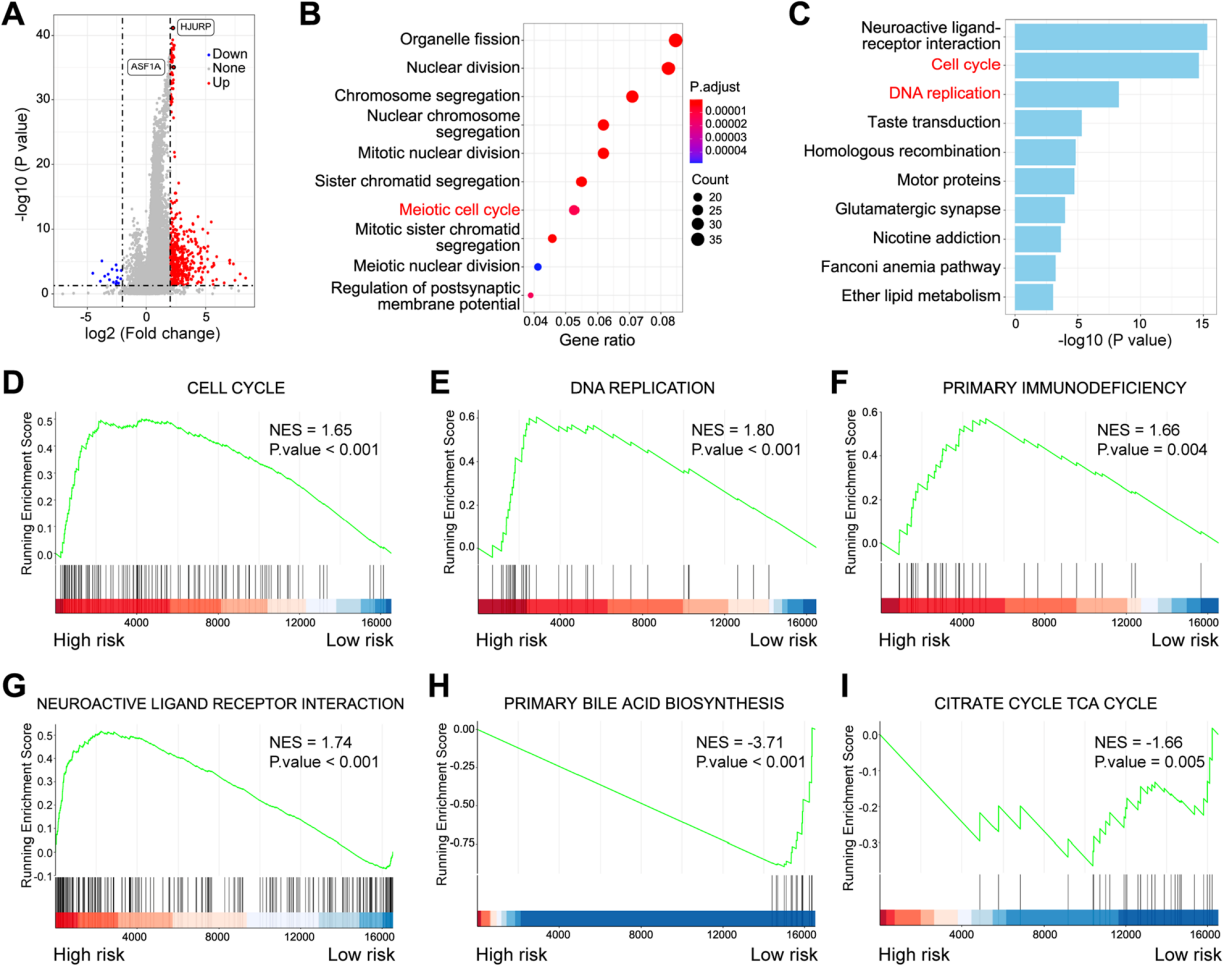
Functional enrichment analysis of different risk groups in TCGA-LIHC. (**A**) Volcano plot of differentially expressed genes between low- and high-risk patients. (**B**) Representative GO terms and pathways enriched from upregulated genes in the high-risk group. (**C**) Representative KEGG terms and pathways enriched from upregulated genes in the high-risk group. Gene set enrichment analysis (GSEA) revealed that genes with higher expression in the high-risk group were enriched in KEGG pathways such as cell cycle (**D**), DNA replication (**E**), primary immunodeficiency (**F**), and neuroactive ligand receptor interaction (**G**), and genes with higher expression in the low-risk group were enriched in KEGG pathways such as primary bile acid biosynthesis (**H**) and citrate cycle TCA cycle (**I**). NES, normalized enrichment score. *p* value < 0.05 was considered statistically significant.

Based on the two-gene model, the samples in the TCGA-LIHC cohort were divided into high-risk and low-risk groups according to the calculated risk scores. The differentially expressed genes (DEGs) between the two groups were identified by the Wilcoxon test, and we observed that 492 genes were upregulated and 16 genes were downregulated in the high-risk group (Fig. 7A). Gene Ontology (GO) term annotation demonstrated that the upregulated genes were mainly involved in organelle fission, nuclear division, chromosome segregation, nuclear chromosome segregation, mitotic nuclear division, sister chromatid segregation, the meiotic cell cycle, and other processes (Fig. 7B). The Kyoto Encyclopedia of Genes and Genomes (KEGG) pathway analysis revealed that the upregulated genes were enriched in the neuroactive ligand‒receptor interaction, cell cycle, DNA replication and other pathways (Fig. 7C). To investigate whether pathway activation is attributed to the direct interaction of ASF1A and HJURP at the regulatory genomic regions of these genes, we checked the ASF1A and HJURP protein feature domains in the UniProt database and drew corresponding schematic diagrams containing the protein feature domains (Supplementary Fig. S8). It was found that neither ASF1A nor HJURP proteins have DNA binding domains, indicating that the two histone chaperones do not directly interact with DNA. Additionally, GSEA results also proved a positive correlation between the high-risk group and cell cycle, DNA replication, primary immunodeficiency, and neuroactive ligand receptor interaction (Fig. 7D–G) and a negative correlation between high risk and primary bile acid biosynthesis and citrate cycle (Fig. 7H,I). Furthermore, increased AFP (alpha-fetoprotein) and bilirubin in the patients of high-risk group were also observed (Supplementary Fig. S9). Taken together, these results suggested that alterations mainly in sustaining proliferation and avoiding immune destruction contribute to the increased risk of HCC based on the two-gene model. Sustaining proliferation is known to be associated with high levels of H3–H4 histone chaperones; however, the relationship between H3–H4 histone chaperones and immune cell infiltration is still unclear in HCC.

### Association between risk score and infiltration immune cells in HCC

**Figure 8:**
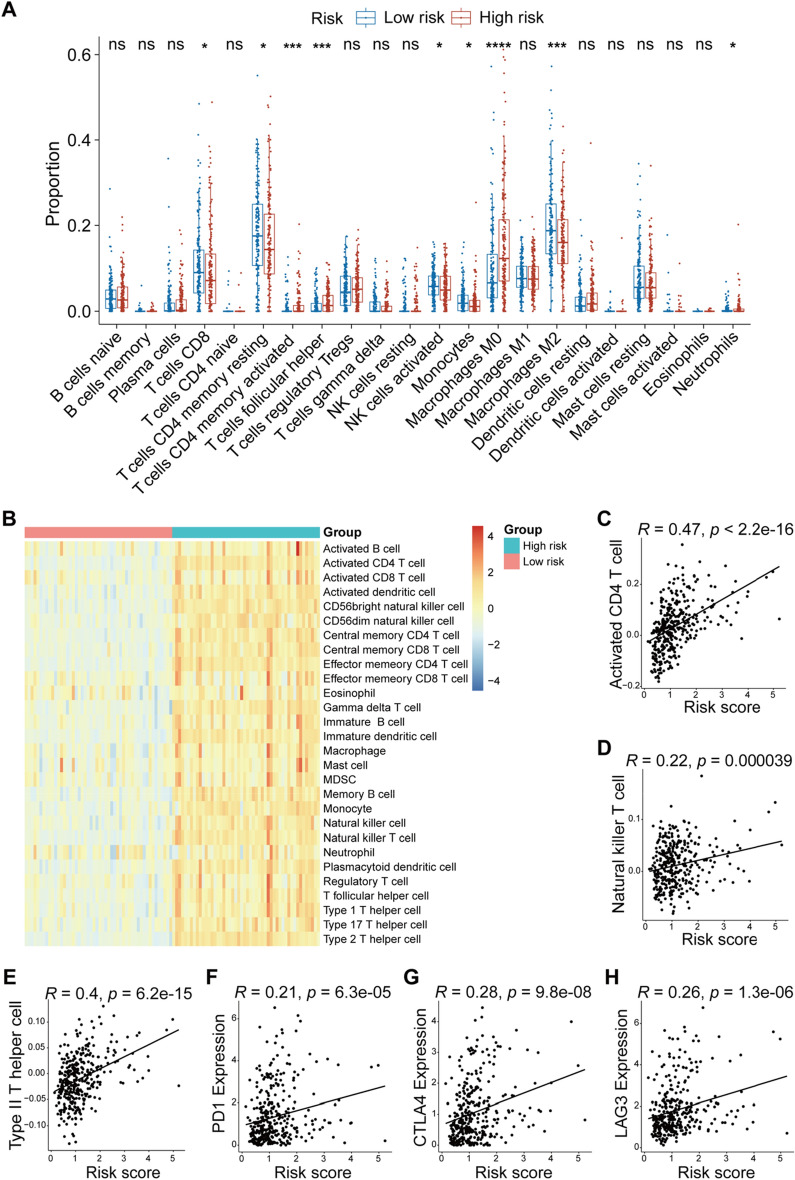
Analysis of the difference in immune infiltration between the high- and low-risk groups. (**A**) The proportions of 22 immune cells in the low- and high-risk groups. (**B**) Heatmap showing the distribution of 28 types of immune cells in HCC patients with the top and bottom 50 risk scores. Spearman’s correlation analysis of the relationship between the risk score and immune cells, including activated CD4 T cells (**C**), natural killer T cells (**D**), and type II T helper cells (**E**). Spearman’s correlation analysis was also conducted to assess the relationship between the risk score and the expression of PD-1 (**F**), CTLA4 (**G**), and LAG3 (**H**). *R* values represent Spearman’s correlation coefficient. The Kruskal‒Wallis test was performed to determine significance in (**A**); ns, not significant; *p < 0.05, ****p < 0.0001.

CIBERSORT was conducted to assess the proportion of immune cells between the high-/low-risk groups. As a result, immune cell populations, including CD8 + T cells, CD4 + memory resting T cells, CD4 + memory activated T cells, follicular helper T cells, activated NK cells, monocytes, M0 macrophages, M2 macrophages, and neutrophils, were found to be significantly different between the two groups (Fig. 8A). We estimated the abundance of 28 kinds of immune cells using ssGSEA based on RNA-seq data from the TCGA-LIHC cohort. We observed more immune cell infiltration in the patients with high risk scores (Fig. 8B). Moreover, the correlation analysis revealed that the risk score was positively correlated with the abundance of activated CD4 T cells (R = 0.47, *p* < 2.2e−16), natural killer T cells (R = 0.22, *p* = 0.000039) and type II T cells (R = 0.4, *p* = 6.2e−15) (Fig. 8C-E). Moreover, we also examined the association of the risk score with multiple predictors of response to immunotherapy. We observed a positive correlation of the risk score with known immune checkpoint genes, including PD-1 (R = 0.21, *p* = 6.3e−05), CTLA4 (R = 0.28, *p* = 9.8e−08) and LAG3 (R = 0.26, *p* = 1.3e−06) (Fig. 8F–H). These results suggested that HCC patients in the high-risk group may be more sensitive to immune checkpoint therapy (ICT).

## Discussion

Liver cancer remains a global health problem with a growing incidence. Hepatocellular carcinoma (HCC) is the most common form of liver cancer and accounts for ~ 90% of cases^1^. Most HCC patients are diagnosed at an advanced stage and resistant to current therapies, resulting in poor prognosis and high mortality. It is necessary to develop effective biomarkers for prognostic and treatment-effect prediction to improve the survival of HCC patients. Previously, the majority of H3–H4 histone chaperones have been identified as tumor-promoting factors through their upregulation or mutation in multiple cancers; however, systematic analysis of H3–H4 histone chaperones is still lacking in HCC^15^. Thus, in this study, we focused on H3–H4 histone chaperones and investigated their predictive effect for prognosis and immune checkpoint therapy in HCC.

### Prognostic roles of H3–H4 histone chaperones and creation of a two-gene model in HCC

Before our study, there were sporadic papers investigating the role of single H3–H4 histone chaperones in HCC^10,16,17^. Increased ASF1A expression is observed in multiple types of cancers, facilitating acetylation of H3K56 in these tumors^18,19^. ASF1B has also been confirmed to promote cell growth^20,21^. Additionally, a previous study suggested that HJURP is an indispensable factor for chromosomal stability in immortalized cancer cells and is a potential novel therapeutic target for the development of anticancer drugs^22^. Xu et al.^17^ reported that CHAF1A may function as a poor prognostic indicator for 5-year overall and disease-free survival in patients with HCC. However, the current work is the first study to provide a comprehensive analysis of the expression level and prognostic value of H3–H4 histone chaperones in HCC. We demonstrated that the expression of 19 known H3–H4 histone chaperones was higher in HCC tumor tissues than in normal liver tissues in the TCGA and GEO datasets (Fig. 2), and KM curves showed that HCC patients with high H3–H4 histone chaperone expression had a shorter survival (Supplementary Fig. S4), but not all of these H3–H4 histone chaperones could be used to predict the prognosis of HCC patients. Thus, univariate Cox analysis was used to identify risk genes associated with patient survival, and we discovered that 15 H3–H4 histone chaperones are risk genes in HCC (Table 2). Given that a single risk gene is not accurate and stable enough to predict the outcome of HCC patients, if all 15 risk genes are included in the construction of a prognostic model, the model will be complicated and redundant, resulting in a lack of clinical practicability. Therefore, we further used LASSO and multivariate Cox to identify important risk genes that can be used for prognosis, and finally, we screened ASF1A and HJURP for the construction of a prognostic model (Fig. 3).

Indeed, in the last decade, bioinformatic analysis tools have improved, and multi-omics data have accumulated rapidly. Many prognostic models for HCC have been developed by using various data sources^23–27^. However, due to the previously limited sample size used to test and validate the models and the lack of validation at the protein level, these models are rarely applied in clinical practice. In this study, first, we constructed a two-gene model (ASF1A and HJURP) using TCGA-LIHC data (346 samples included) as a training set. The model was then evaluated with ICGC-LIRI (230 samples included) and GSE14520 (a prevailing dataset in HCC study, 209 samples included) as the testing sets, and we identified that the model has high sensitivity and specificity for predicting the outcome of HCC patients (1-, 3-, and 5-year AUC of 0.767, 0.731, and 0.809, respectively, in ICGC-LIRI; 1-, 3-, and 5-year AUC of 0.782, 0.782, and 0.732, respectively, in GSE14520) (Fig. 4). We compared our two-gene model with other previous HCC prognostic models^28,29^, and the performance of our model is comparable with previously published models by comparing the areas of under the curve (AUCs) among models to predict 1-, 3-, and 5-year survival in HCC patients. In addition, we used prognostic information-containing tissue microarray data as a validation set to verify the model at the protein level. Although ASF1A or HJURP alone could not significantly distinguish the prognosis of patients (Fig. 6G,H), the two-gene model could effectively predict the prognosis of HCC patients (Fig. 6F), which also indicated the stability and reliability of the model in predicting the prognosis of HCC patients. Taken together, the two-gene model passes the examination with nearly 800 samples and is validated at the protein level. Given that immunohistochemical staining assays are readily accessible in many hospitals, this two-gene model may have promising clinical applicability.

### Signaling pathways involving this two-gene model in HCC

Why can the two-gene model be used to predict the prognosis of HCC patients? Either the two genes are the key hinges of their involved signaling pathways, or the abnormal expression of these two genes reflects changes in the activity of related signaling pathways in HCC. Therefore, we explored the signalling pathways underlying the two-gene model, divided HCC patients into high- and low-risk groups according to the risk scores obtained by the model, and performed differential expression gene analysis, GO, KEGG, and GSEA. We observed that the cell cycle and DNA replication were significantly activated in high-risk patients (Fig. 7B–E), which is consistent with previous findings^30,31^. In addition, we also identified changes in other signaling pathways in high-risk patients, such as primary immunodeficiency, neuroactive ligand receptor interaction, primary bile acid biosynthesis, TCA circulation, and primary immune deficiency (Fig. 7F–I).

### The potential application of two-gene models to predict immunotherapy outcomes offers hope for HCC patients

Previously, HJURP was reported to be associated with tumor-infiltrating immune cells, immune checkpoints, and immune suppression in HCC^32,33^. Moreover, in this study, we found that a high risk score was positively correlated with immune deficiency, suggesting that immune dysfunction may exist in high-risk patients (Fig. 7F). To investigate whether the two-gene model can predict the level of immune infiltration in HCC patients and provide guidance for immunotherapy, we evaluated the level of immune cell infiltration in HCC patients by CIBERSORT and ssGSEA and observed a higher proportion of immune cells in high-risk patients (Fig. 8A,B). In addition, we identified a positive correlation of the risk score with the expression of PD1, CTLA4, and LAG3 (Fig. 8F–H), suggesting that high-risk patients may be more sensitive to these immune checkpoint therapies.

In summary, we developed a two-gene model based on H3–H4 histone chaperones that can not only predict the survival outcome of HCC patients but also evaluate the levels of immune cell infiltration in HCC tissues to assess immunotherapy sensitivity for patients with HCC.

### Prospect of the study

Future applications of the two-gene model are considered to accurately predict the prognosis of HCC patients and distinguish patients sensitive to ICT immunotherapy, which is beneficial for improving the survival rate, treatment accuracy and quality of life of HCC patients.

### Limitations of the study

Although our study demonstrated that the two-gene model is effective for predicting survival outcomes and sensitivity to immunotherapy for HCC patients, several issues remain. First, for a prevailing prognostic model in the clinic, nearly 800 samples are still not enough, and more extensive clinical data are needed for further validation. The data utilized in this study encompassed transient gene expression patterns of patients at a specific point in time, thereby lacking the capacity to capture the dynamic characteristic changes associated with tumor progression. Additionally, the importance of H3–H4 histone chaperones in their involved signaling pathways also needs to be explored in vivo and in vitro.

## Supplementary Information

Supplementary Information 1.

Supplementary Information 2.

Supplementary Information 3.

## Data Availability

The data generated in this study are included in the supplementary material. Further inquiries can be directed to the corresponding author. In this study, RNA-Seq data and corresponding clinical characteristics were obtained from the TCGA database (https://www.cancer.gov/ccg/research/genome-sequencing/tcga, accessed on 3 January 2023) and International Cancer Genome Consortium (ICGC) database (https://dcc.icgc.org/, accessed on 3 January 2023). The GSE121248, GSE33006, and GSE14520 datasets were downloaded from the Gene Expression Omnibus (https://www.ncbi.nlm.nih.gov/geo/, accessed on 3 January 2023). The absolute abundance of immune and stromal cell expression profiles was obtained from CIBERSORT (http://CIBERSORT.stanford.edu/, accessed on 3 January 2023).

## Abbreviations

AUC: Area under curve
C-index: Concordance index
DEGs: Differentially expressed genes
GEO: Gene Expression Omnibus
GO: Gene ontology
GSEA: Gene set enrichment analysis
GSVA: Gene set variation analysis
HCC: Hepatocellular carcinoma
ICGC: International Cancer Genome Consortium
ICGC-LIRI: International Cancer Genome Consortium-Liver cancer-RIKEN
IHC: Immunohistochemistry
KEGG: Kyoto Encyclopedia of Genes and Genomes
KM: Kaplan‒Meier
LASSO: Least absolute shrinkage and selection operator
OS: Overall survival
ROC: Receiver operating characteristic
ssGSEA: Single sample gene set enrichment analysis
TCGA-LIHC: The Cancer Genome Atlas-Liver hepatocellular carcinoma
TMAs: Tissue microarrays
