# Author Correction: Identification of ASF1A and HJURP by global H3–H4 histone chaperone analysis as a prognostic two-gene model in hepatocellular carcinoma

**DOI:** 10.1038/s41598-024-71753-0

**Published:** 2024-09-05

**Authors:** Yongkang Liu, Shihui Liu, Rui Jing, Congcong Li, Yongqi Guo, Zhiye Cai, Pei Xi, Penggao Dai, Lintao Jia, Hongli Zhu, Xiang Zhang

**Affiliations:** 1 National Engineering Research Center for Miniaturized Detection Systems, College of Life Science, Northwest University, Xi’an, 710069 Shaanxi China; 2 State Key Laboratory of Holistic Integrative Management of Gastrointestinal Cancers, Department of Biochemistry and Molecular Biology, Fourth Military Medical University, Xi’an, 710032 Shaanxi China; 3 The Ministry of Education Key Lab of Hazard Assessment and Control in Special Operational Environment, Fourth Military Medical University, Xi’an, 710032 Shaanxi China

Correction to: *Scientific Reports* 10.1038/s41598-024-58368-1, published online 01 April 2024

In the original version of this Article, Hongli Zhu was omitted as a corresponding author. Correspondence and requests for materials should also be addressed to zhuyjw1971@nwu.edu.cn.

The original Article has been corrected.

