# Supplementary material for: Identification of ASF1A and HJURP by global H3–H4 histone chaperone analysis as a prognostic two-gene model in hepatocellular carcinoma
Source: Sci Rep. 2024 Apr 1;14:7666. doi: 10.1038/s41598-024-58368-1 (PMC10984954; doi:10.1038/s41598-024-58368-1)
Supplement: Supplementary file 2 — Supplementary Information 2. [file 41598_2024_58368_MOESM2_ESM.pptx]

## Slide 1
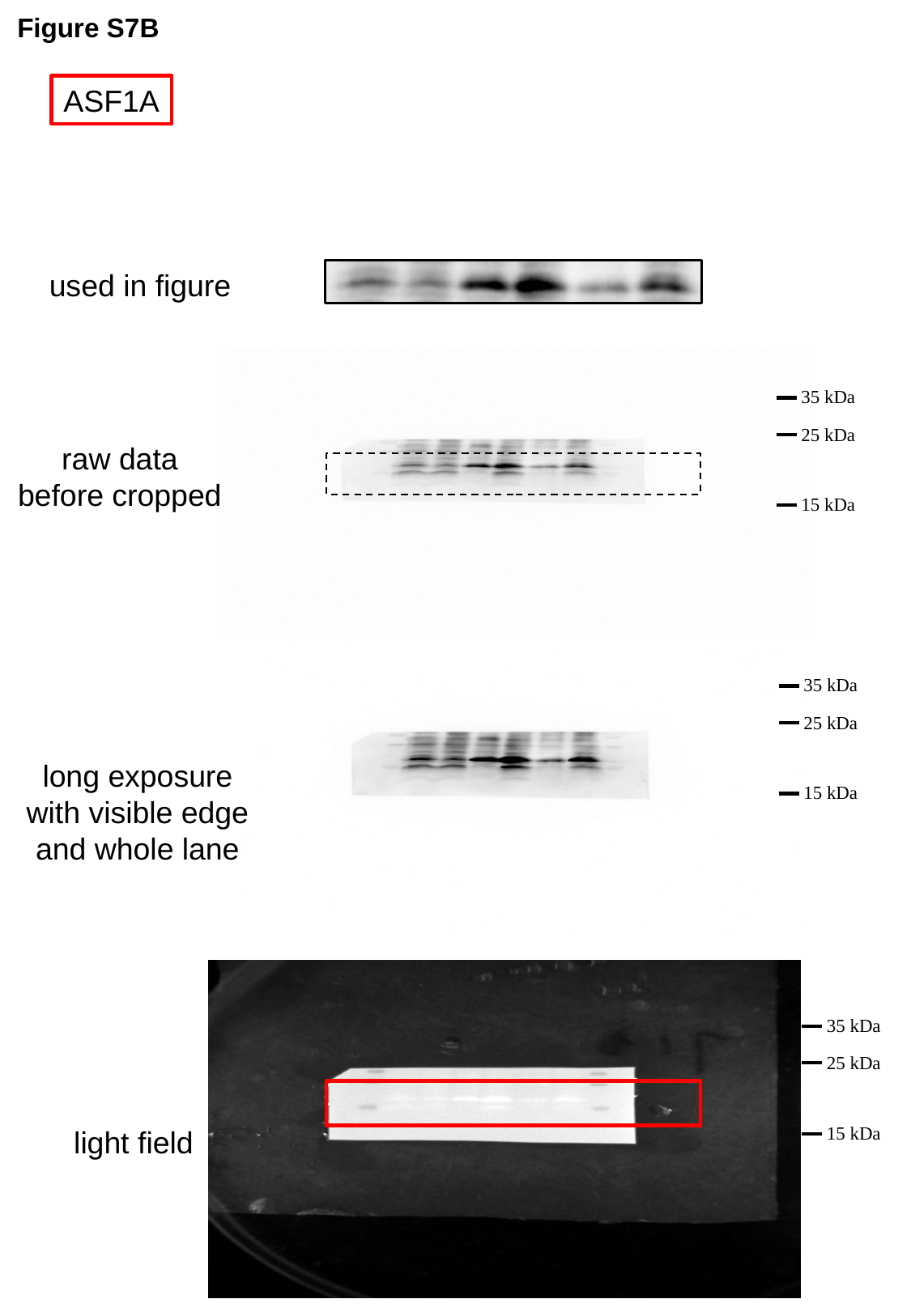

Figure S7B
ASF1A
used in figure
35 kDa
25 kDa
raw data
before cropped
15 kDa
35 kDa
25 kDa
long exposure
with visible edge
and whole lane
15 kDa
35 kDa
25 kDa
15 kDa
light field

## Slide 2
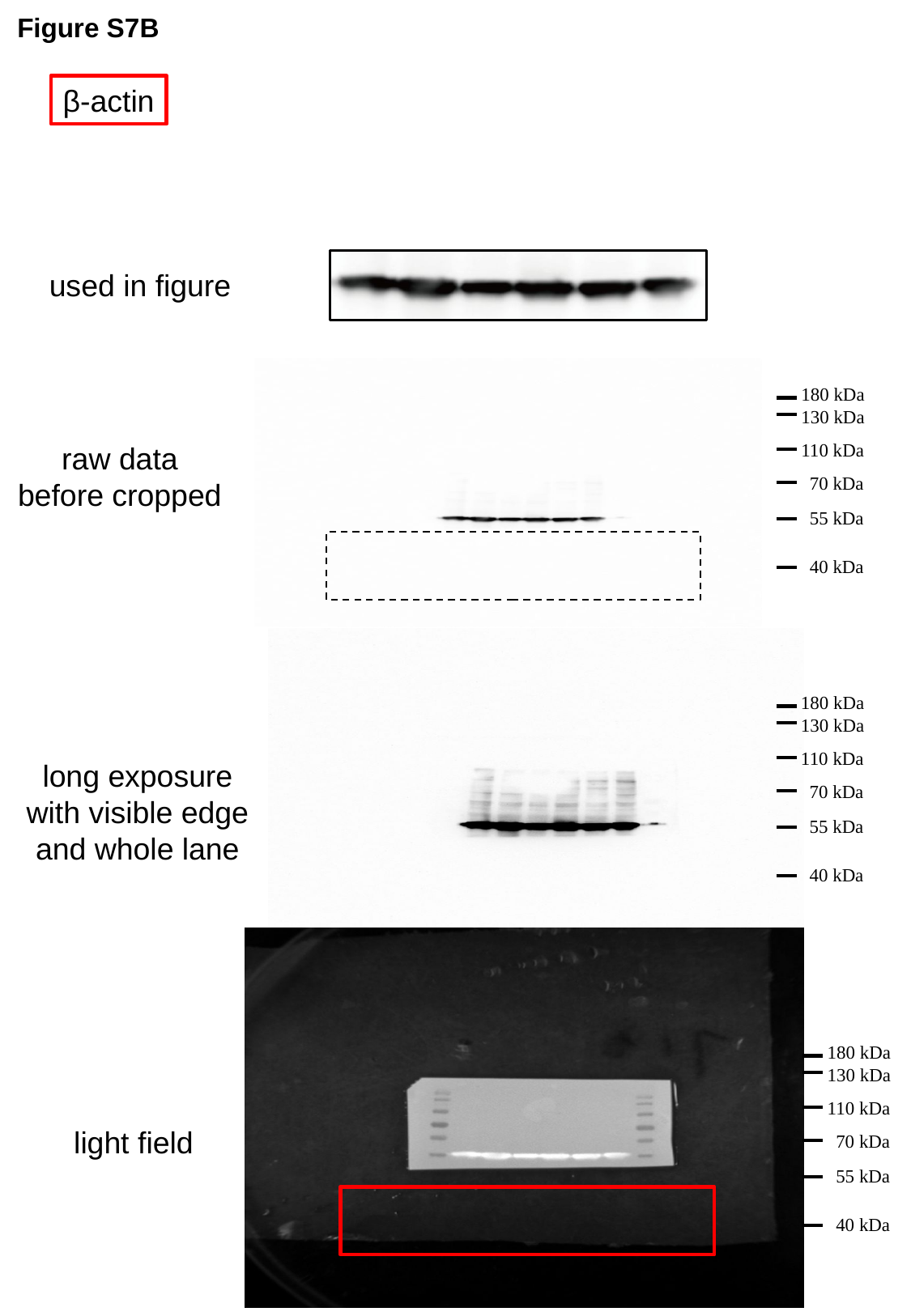

Figure S7B
β-actin
used in figure
180 kDa
130 kDa
110 kDa
raw data
before cropped
70 kDa
55 kDa
40 kDa
180 kDa
130 kDa
110 kDa
long exposure
with visible edge
and whole lane
70 kDa
55 kDa
40 kDa
180 kDa
130 kDa
110 kDa
light field
70 kDa
55 kDa
40 kDa

## Slide 3
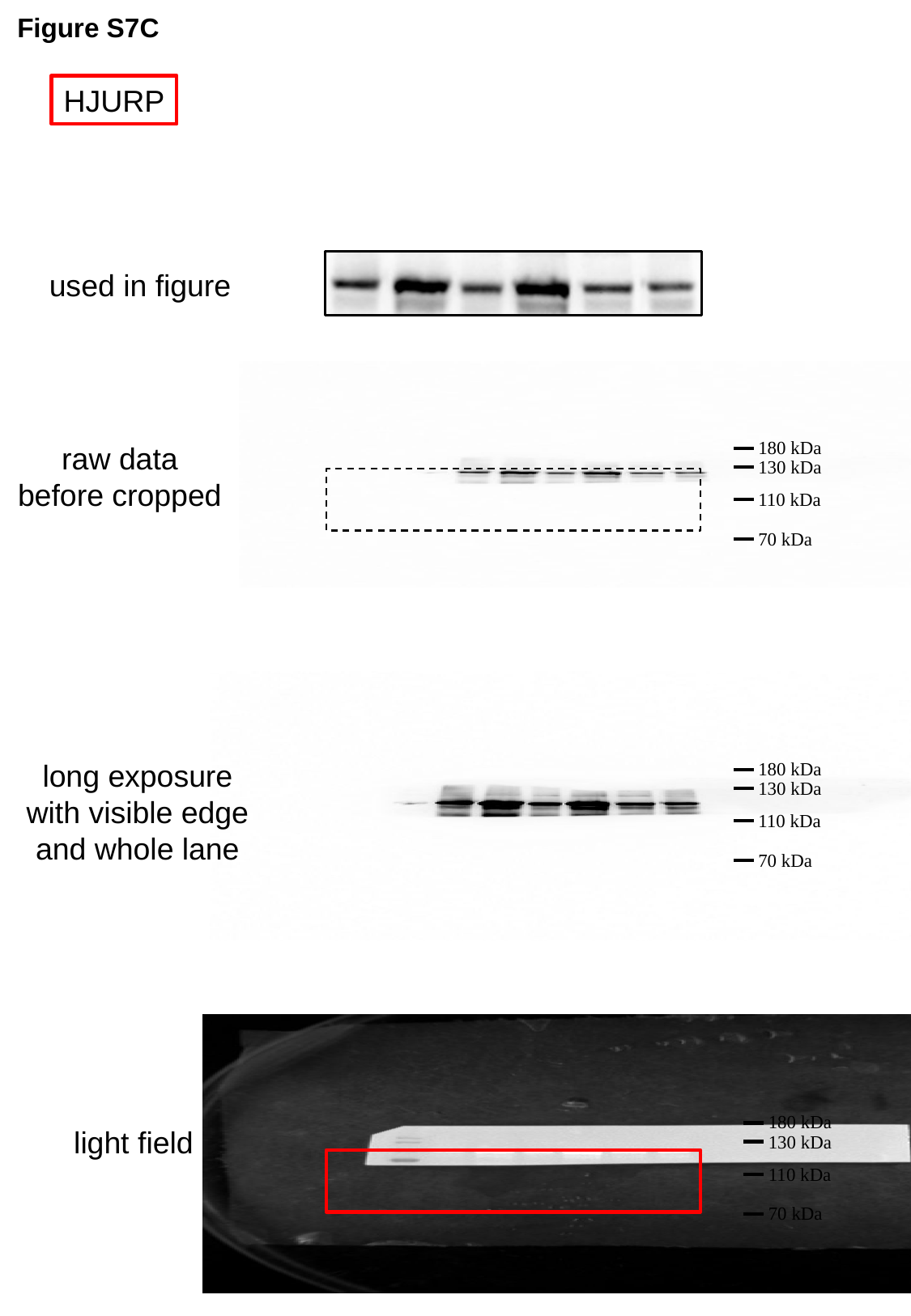

Figure S7C
HJURP
used in figure
180 kDa
raw data
before cropped
130 kDa
110 kDa
70 kDa
long exposure
with visible edge
and whole lane
180 kDa
130 kDa
110 kDa
70 kDa
180 kDa
light field
130 kDa
110 kDa
70 kDa

## Slide 4
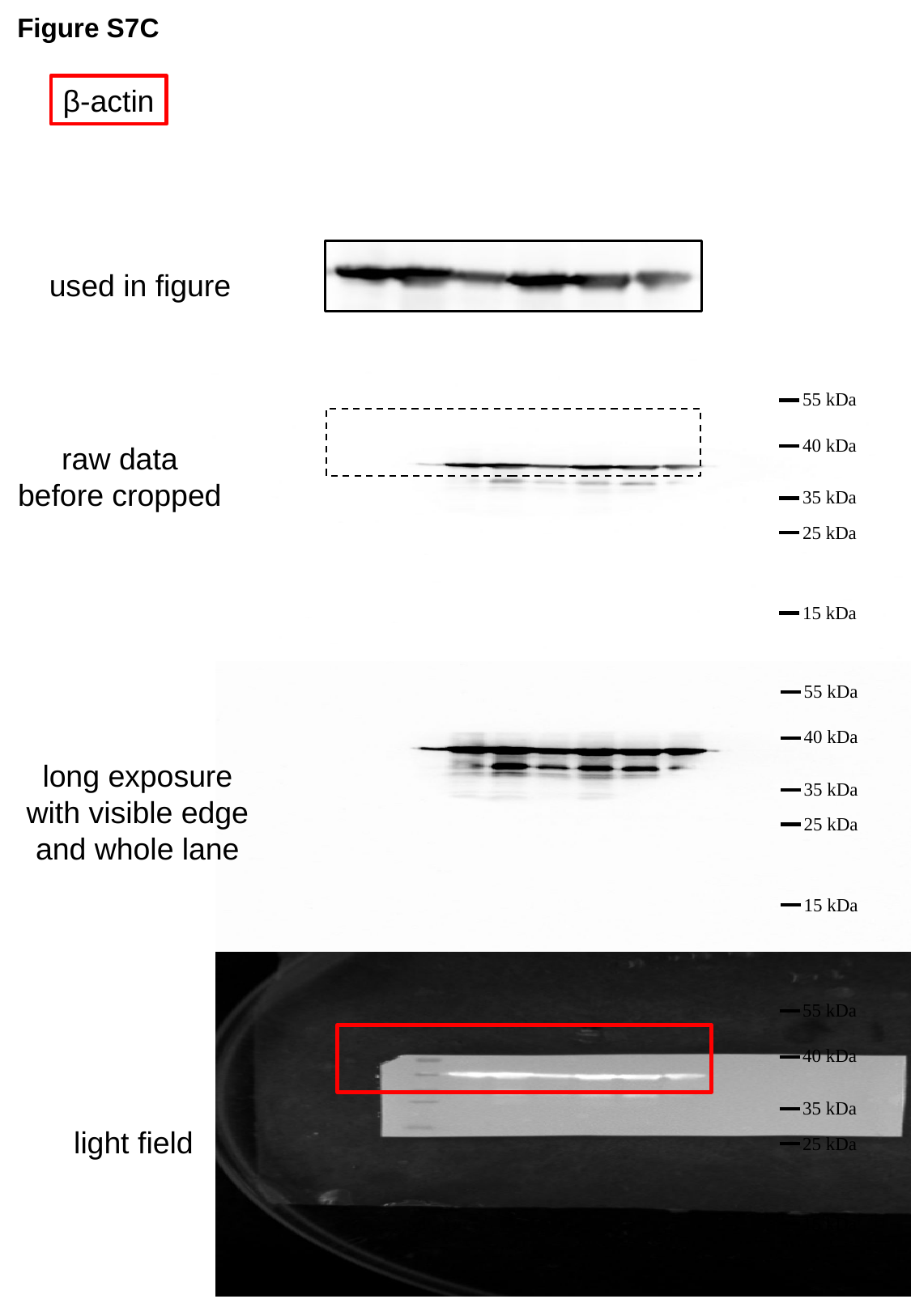

Figure S7C
β-actin
used in figure
55 kDa
40 kDa
raw data
before cropped
35 kDa
25 kDa
15 kDa
55 kDa
40 kDa
long exposure
with visible edge
and whole lane
35 kDa
25 kDa
15 kDa
55 kDa
40 kDa
35 kDa
light field
25 kDa
15 kDa
