# Supplementary material for: Identification of ASF1A and HJURP by global H3–H4 histone chaperone analysis as a prognostic two-gene model in hepatocellular carcinoma
Source: Sci Rep. 2024 Apr 1;14:7666. doi: 10.1038/s41598-024-58368-1 (PMC10984954; doi:10.1038/s41598-024-58368-1)
Supplement: Supplementary file 3 — Supplementary Information 3. [file 41598_2024_58368_MOESM3_ESM.pdf]

Supplementary Material

Identification of ASF1A and HJURP by Global H3-H4 Histone Chaperone Analysis as a Prognostic Two-gene Model in Hepatocellular Carcinoma

Yongkang Liu[1,2], Shihui Liu[1], Rui Jing[1,2], Congcong Li[1], Yongqi Guo[1], Zhiye Cia[1], Pei Xi[1], Penggao Dai[1], Lintao Jia[2], Shuli Cheng[1,4], Hongli Zhu[1,*] and Xiang Zhang[2,3,*]

*** Correspondence:** Xiang Zhang: biozx@fmmu.edu.cn

1 Supplementary Figures and Tables

1.1 Supplementary Figures

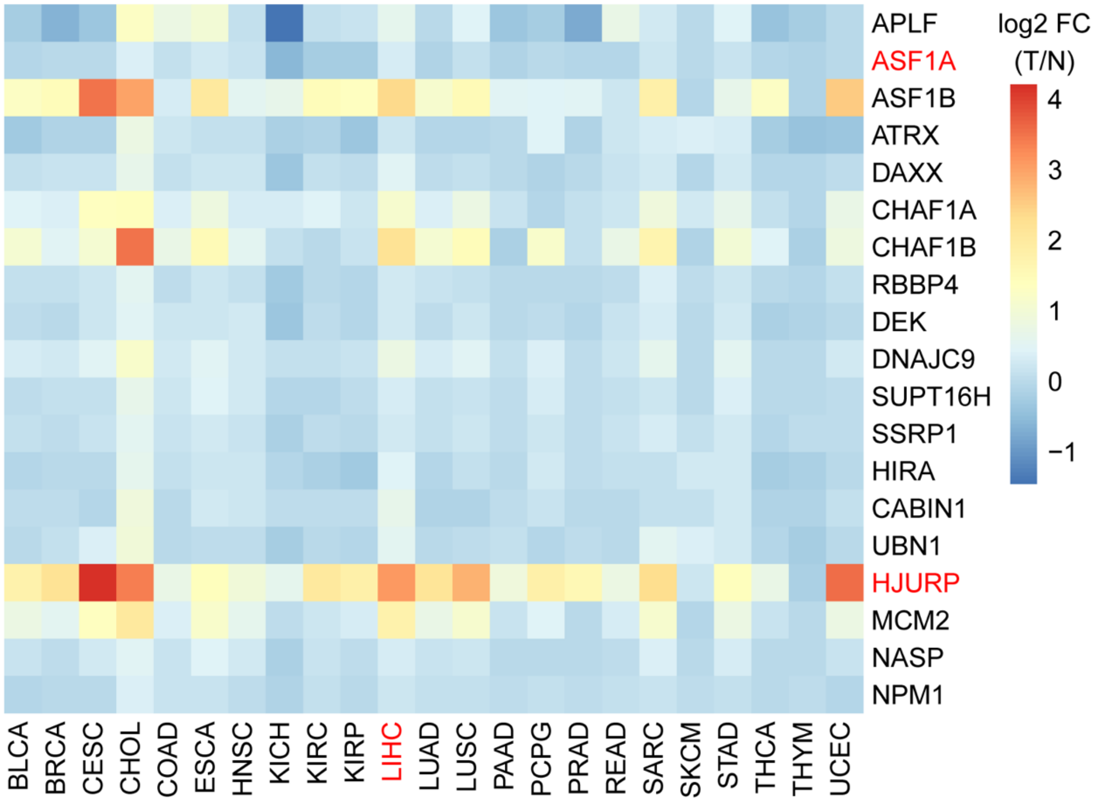

Supplementary Figure S1. The expression differences in histone chaperones between tumor and normal tissues across cancers. FC, Fold Change; T/N, Tumor/Normal.

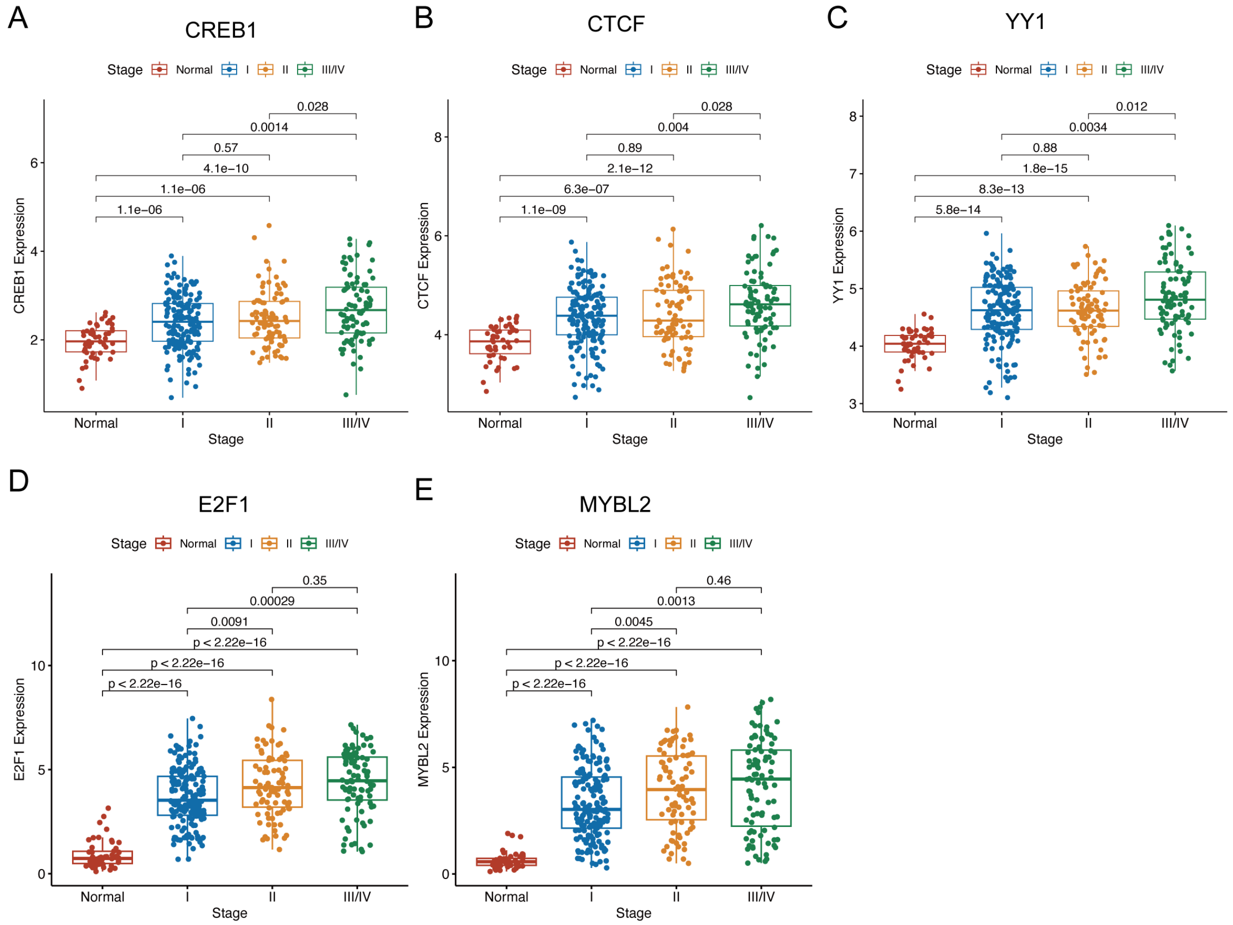

Supplementary Figure S2. The expression changes of potential transcription factors of H3-H4 histone chaperones with tumor progression in TCGA-LIHC. Statistical significance was computed using the Kruskal-Wallis test.

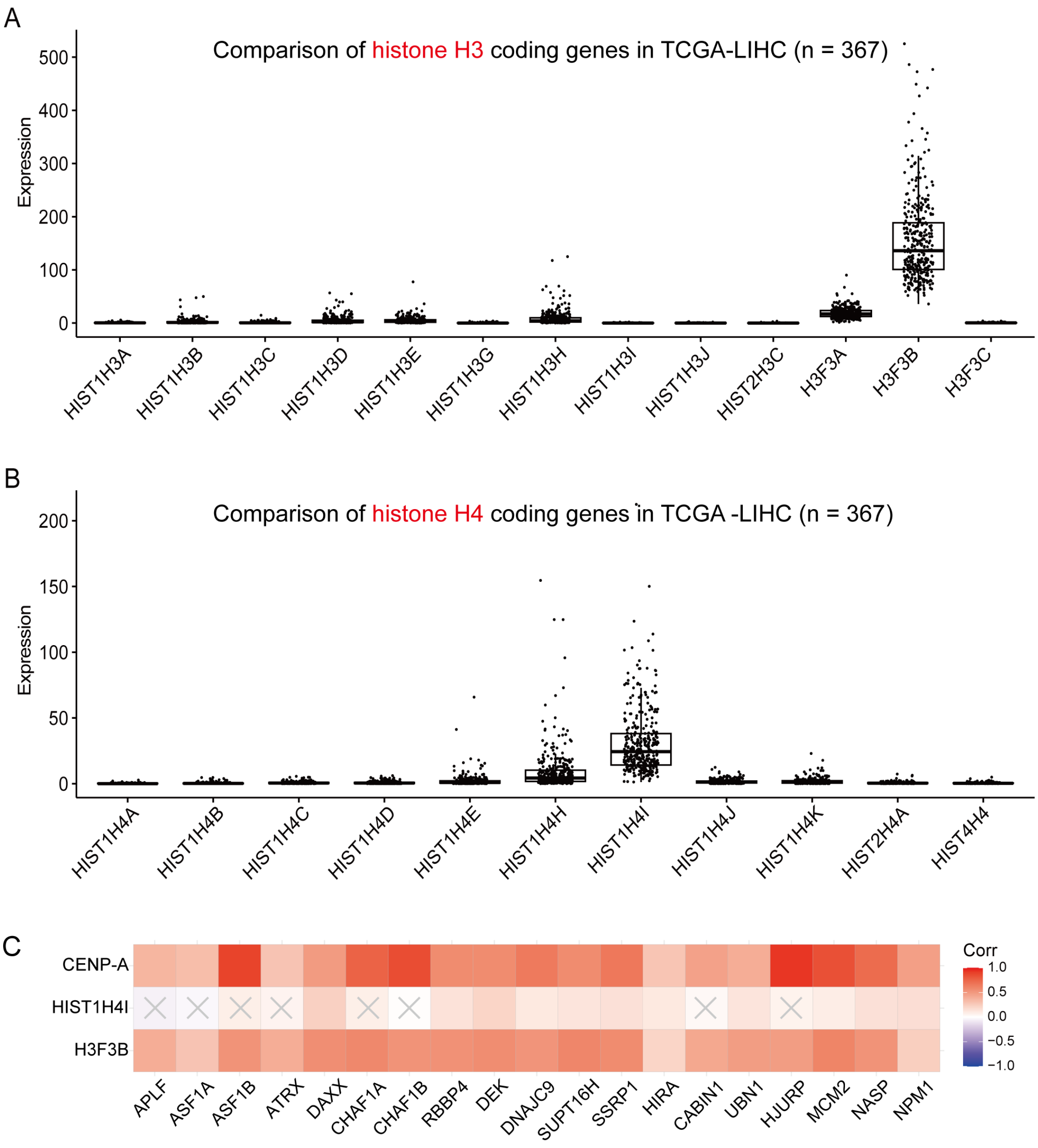

Supplementary Figure S3. Comparison of histone H3 **(A)** and histone H4 **(B)** coding genes in TCGA-LIHC; **(C)** Heatmap of correlation between the expression of histone H3, H4, CENP-A and histone chaperones. Corr, correlation coefficient; "×" means p-value < 0.05.

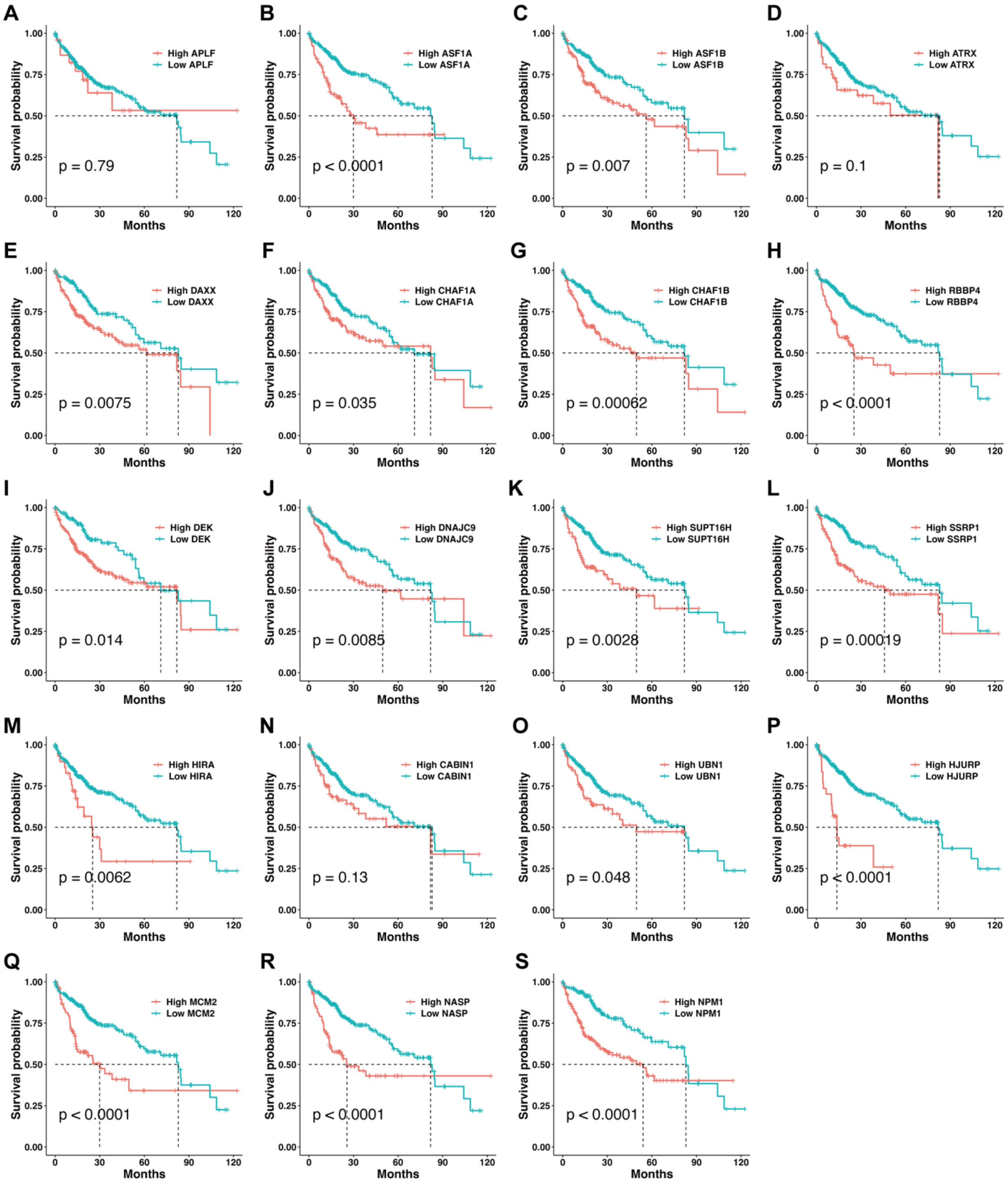

Supplementary Figure S4. Kaplan-Meier (KM) survival analysis of each H3-H4 histone chaperone in hepatocellular carcinoma.

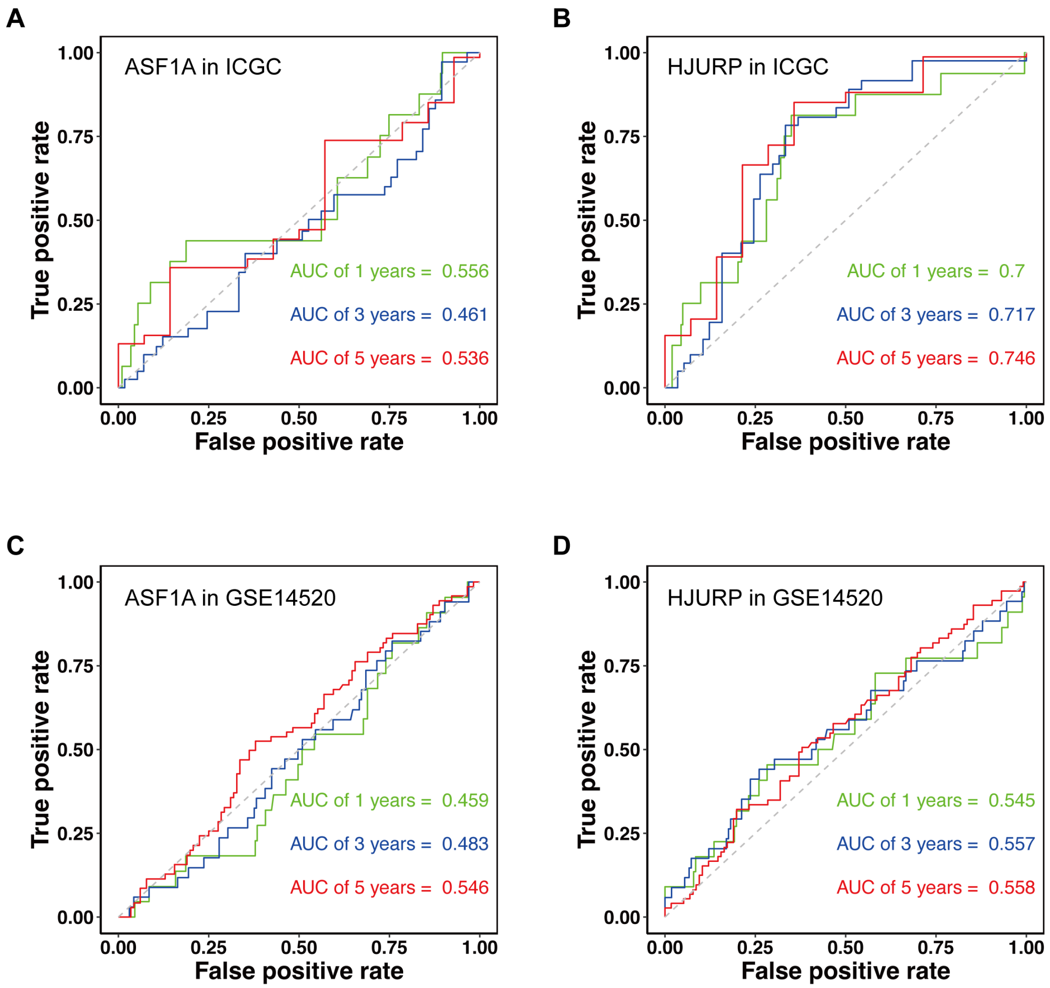

Supplementary Figure S5. The AUC values for ASF1A or HJURP in predicting 1, 3, and 5-year survival rates among HCC patients in ICGC **(A, B)** and GSE14520 **(C, D)** datasets.

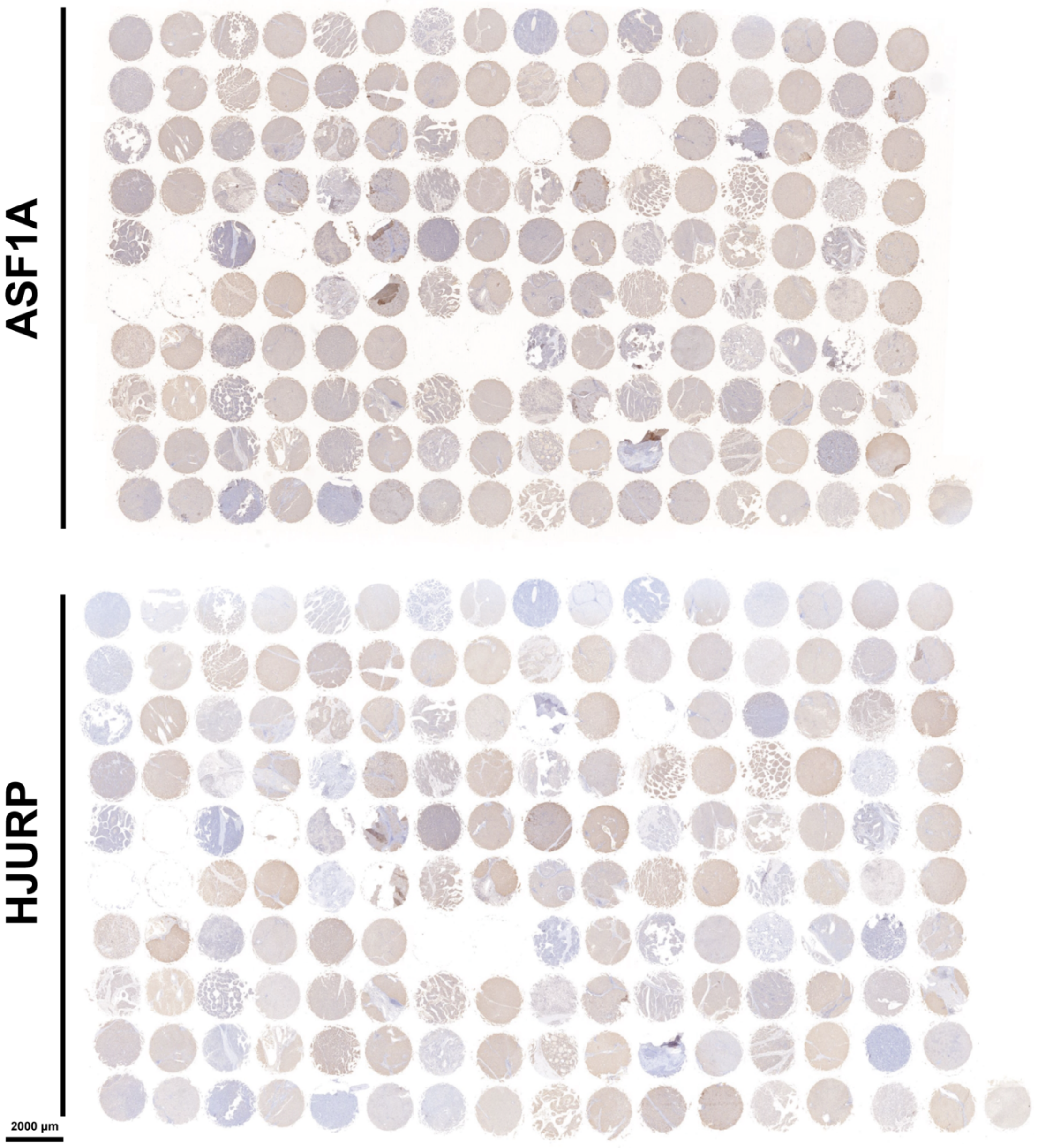

Supplementary Figure S6. Scan images of IHC for ASF1A and HJURP in HCC tissue microarrays.

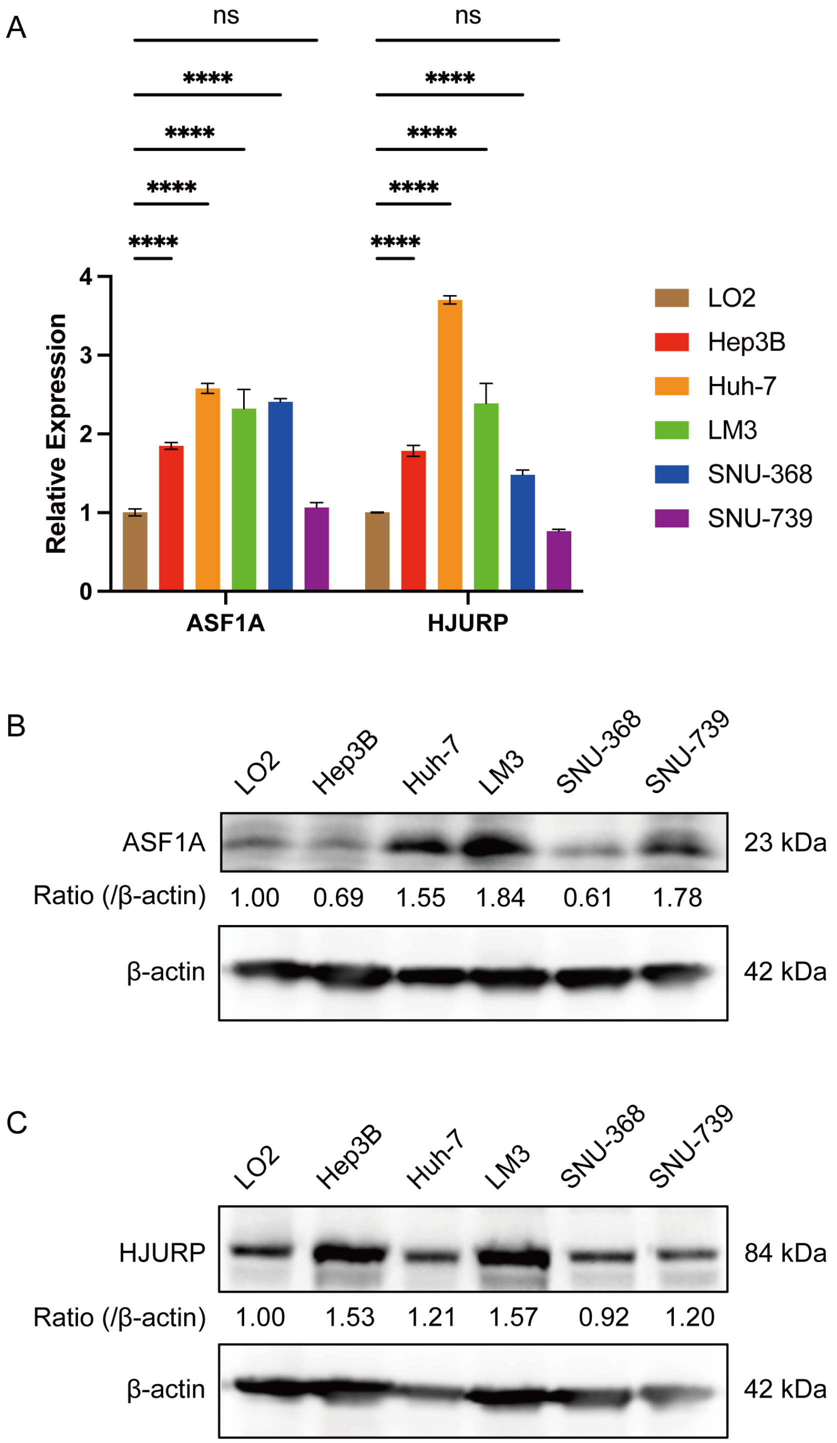

Supplementary Figure S7. (A) The relative expression levels of ASF1A and HJURP were detected at the RNA level by qRT-PCR. The relative expression levels of ASF1A **(B)** and HJURP **(C)** were detected at the protein level by western blotting. Statistical significance was computed using ordinary one-way ANOVA (Figure S6A); ns, not significant; ****, $p < 0.0001$.

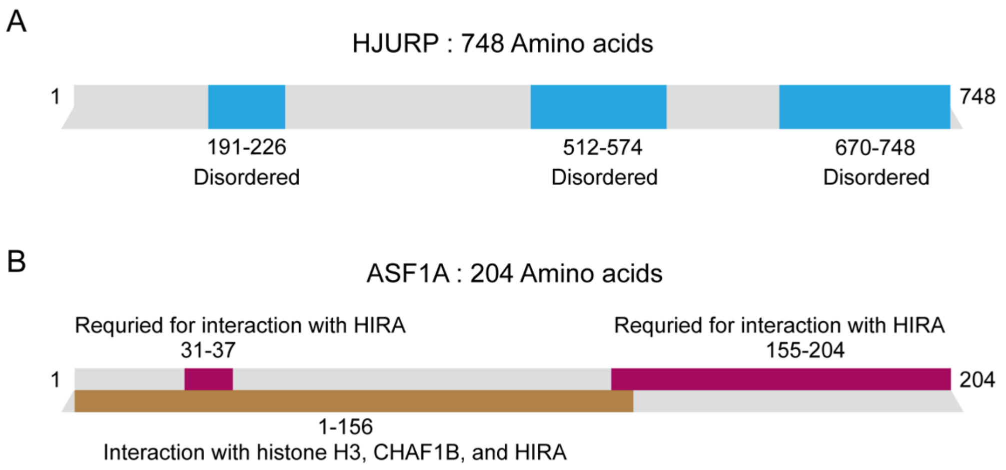

Supplementary Figure S8. The feature domains exist in the ASF1A and HJURP proteins from the UniPort database.

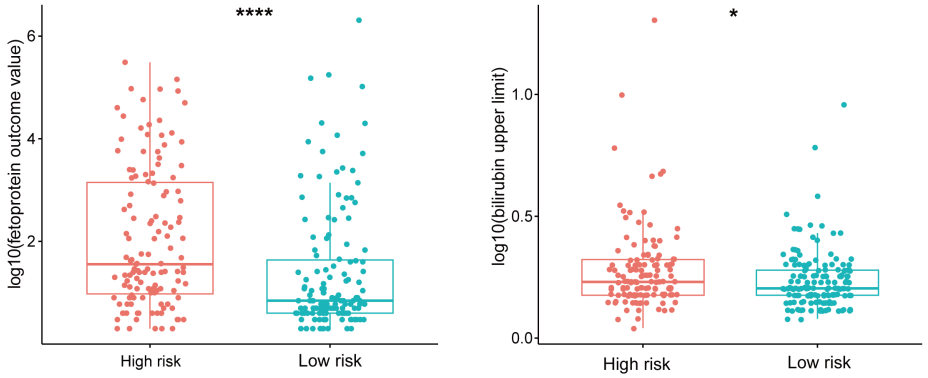

Supplementary Figure S9. Patients in high-risk group have higher AFP expression and bilirubin levels than patients in low-risk group. Data were from TCGA-LIHC.

1.2 Supplementary Tables

Supplementary Table S1. Clinicopathological characteristics of 62 HCC patients

Patient NO.	Gender	Age	Stage	Location in liver	Pathology
1	Male	39	II	right lobe	Highly differentiated HCC
2	Male	63	I	right lobe	Moderately differentiated HCC
3	Male	70	II	right lobe	Moderately differentiated HCC
4	Male	70	I	liver	Highly differentiated HCC
5	Male	52	II	liver	Moderately differentiated HCC
6	Male	44	II	liver	Moderately differentiated HCC
7	Female	52	II	superior segment of right anterior lobe	Moderately differentiated HCC
8	Male	59	II	liver	Moderately differentiated HCC
9	Male	41	I	left lobe	Highly differentiated HCC
10	Male	71	II	right lobe	Highly/Moderately differentiated HCC
11	Male	61	II	liver	Moderately differentiated HCC
12	Male	48	II	liver	Moderately differentiated HCC
13	Male	41	II	liver	Moderately differentiated HCC

14	Male	59	II	right posterior lobe	HCC
15	Male	64	II	right lobe	Moderately differentiated HCC
16	Female	64	II	right lobe	HCC
17	Male	43	III	right lobe	HCC
18	Male	34	III	liver	HCC
19	Male	54	I	liver	HCC
20	Male	34	II	right posterior lobe	HCC
21	Male	53	I	liver	HCC
22	Male	53	II	liver	Moderately differentiated HCC
23	Male		II	right lobe	HCC
24	Male	34	II	left lobe	HCC
25	Female	36	II	right lobe	Moderately differentiated HCC
26	Female	31	II	right lobe	Moderately differentiated HCC
27	Male	55	II	left lobe	Moderately differentiated HCC
28	Male	67	II	right lobe	Moderately differentiated HCC
29	Male	51	II	caudate lobe	Moderately differentiated HCC
30	Female	51	III	liver	Moderately/Poorly differentiated HCC
31	Male	66	II	middle lobe	Moderately differentiated HCC
32	Male	44	III	left lobe	Moderately/Poorly differentiated HCC
33	Male	54	II	liver	Moderately differentiated HCC
34	Female	72	III	liver	Poorly differentiated HCC
35	Male	65	I	liver	Highly differentiated HCC
36	Male	65	II	liver	HCC
37	Male	70	I	liver	Highly differentiated HCC
38	Male	43	II	liver	Moderately differentiated HCC
39	Male	33	III	left lobe	Poorly differentiated HCC
40	Male	52	III	liver	Poorly differentiated HCC
41	Male	55	II	liver	Moderately differentiated HCC
42	Male	58	III	liver	Moderately differentiated HCC
43	Male	58	II	liver	Moderately/Poorly differentiated HCC
44	Male	50	II	liver	Moderately differentiated HCC
45	Male	27	II	liver	Moderately differentiated HCC
46	Female	55	II	left lobe	Poorly differentiated HCC
47	Male	73	II	right anterior lobe	Highly/Moderately differentiated HCC
48	Male	54	I	right lobe	Highly differentiated HCC
49	Male	44	III	right lobe	Moderately/Poorly differentiated HCC
50	Male	59	II	right femur	Moderately differentiated HCC
51	Male	72	I	liver	Highly differentiated HCC
52	Female	38		liver	HCC
53	Male	48	II	liver	HCC
54	Male	45	II	liver	Moderately differentiated HCC
55	Female		II	liver	Highly/Moderately differentiated HCC
56			I	right lobe	Highly differentiated HCC
57	Male	63	II	left lobe	Moderately differentiated HCC
58	Male	46	III	liver	HCC
59	Male	66	I	liver	Highly differentiated HCC
60	Male	45	I	right lobe	Highly differentiated HCC
61	Male	58	I	liver	Highly differentiated HCC
62	Male	48	I	liver	Highly/Moderately differentiated HCC

Supplementary Table S2. Antibodies used for immunohistochemical (IHC) and Western blotting

No.	Antibody name	Host/Isotype	Reactivity	Company	Cat No.	Dilution
1	ASF1A Polyclonal antibody	Rabbit/IgG	Human ASF1A	Proteintech	10784-1-AP	1:50 (IHC)<br>1:1000 (WB)
2	HJURP Polyclonal antibody	Rabbit/IgG	Human HJURP	Proteintech	15283-1-AP	1:200 (IHC)<br>1:1000 (WB)

Supplementary Table S3. Primers used for qRT-PCR

Target	Primer sequence (5' to 3')
ASF1A	Forward: TGCTGGATAACCCTTCTCCTT
	Reverse: CAGGACCCACTAAAACAGAGTC
HJURP	Forward: CCACGCTGACCTACGAGAC
	Reverse: CTCACCGCTTTTTGAATCGGC
GAPDH	Forward: CTGGGCTACACTGAGCACC
	Reverse: AAGTGGTCGTTGAGGGCAATG

Supplementary Table S4. ChEA3 predicted 23 transcription factors regulating more than 10 H3-H4 histone chaperones

TF	Overlapping_Genes	Count
E2F4	DAXX,NPM1,SUPT16H,HJURP,SSRP1,DEK,HIRA,CHAF1B,CHAF1A,NASP,RBBP4,APLF,DNAJC9,ASF1B,MCM2	15
CTCF	NPM1,SUPT16H,ATRX,HJURP,DEK,SSRP1,CABIN1,HIRA,CHAF1B,CHAF1A,RBBP4,NASP,DNAJC9,ASF1A,ASF1B	15
E2F1	SUPT16H,NPM1,HJURP,DEK,SSRP1,CABIN1,HIRA,CHAF1B,CHAF1A,NASP,RBBP4,DNAJC9,ASF1B,MCM2	14
MYC	NPM1,DAXX,SUPT16H,ATRX,DEK,SSRP1,CABIN1,HIRA,RBBP4,NASP,DNAJC9,MCM2,ASF1B	13
KDM5B	SUPT16H,NPM1,ATRX,HJURP,DEK,CABIN1,HIRA,CHAF1A,NASP,RBBP4,UBN1,ASF1A,ASF1B	13
CREM	DAXX,SUPT16H,NPM1,SSRP1,DEK,CABIN1,RBBP4,NASP,APLF,DNAJC9,ASF1A,MCM2,ASF1B	13
FOXM1	NPM1,SUPT16H,HJURP,DEK,SSRP1,HIRA,CHAF1B,CHAF1A,NASP,DNAJC9,ASF1B,MCM2	12
SETDB1	HIRA,DAXX,NPM1,CHAF1A,RBBP4,NASP,UBN1,DNAJC9,SSRP1,DEK,CABIN1,MCM2	12
JUN	HIRA,NPM1,APLF,UBN1,HJURP,DNAJC9,SSRP1,DEK,ASF1A,MCM2,CABIN1,ASF1B	12
TFDP1	HIRA,NPM1,CHAF1B,CHAF1A,NASP,HJURP,DNAJC9,DEK,SSRP1,ASF1B,MCM2	11
E2F8	CHAF1B,SUPT16H,NPM1,CHAF1A,NASP,HJURP,DNAJC9,DEK,SSRP1,MCM2,ASF1B	11
MAZ	HIRA,DAXX,NPM1,CHAF1A,NASP,HJURP,DNAJC9,DEK,SSRP1,CABIN1,MCM2	11
GATA1	HIRA,NPM1,CHAF1B,NASP,APLF,HJURP,DNAJC9,SSRP1,ASF1A,ASF1B,CABIN1	11
SP1	HIRA,DAXX,NPM1,CHAF1B,CHAF1A,NASP,UBN1,HJURP,DNAJC9,CABIN1,ASF1B	11
MITF	HIRA,NPM1,DAXX,SUPT16H,CHAF1A,RBBP4,ATRX,UBN1,MCM2,CABIN1,ASF1B	11
IRF3	HIRA,NPM1,CHAF1A,RBBP4,NASP,ATRX,UBN1,HJURP,ASF1B,MCM2,CABIN1	11
ZNF367	NPM1,CHAF1B,CHAF1A,NASP,HJURP,DNAJC9,DEK,SSRP1,ASF1B,MCM2	10
MYBL2	HIRA,CHAF1B,NPM1,CHAF1A,NASP,HJURP,SSRP1,ASF1A,ASF1B,MCM2	10
YY1	HIRA,DAXX,NPM1,CHAF1A,RBBP4,ATRX,HJURP,DEK,SSRP1,MCM2	10
TCF3	HIRA,SUPT16H,NPM1,CHAF1A,RBBP4,NASP,HJURP,SSRP1,ASF1B,MCM2	10
ELK1	HIRA,DAXX,RBBP4,NASP,APLF,HJURP,DEK,SSRP1,ASF1A,CABIN1	10
TEAD4	HIRA,CHAF1B,CHAF1A,RBBP4,APLF,DNAJC9,SSRP1,ASF1A,ASF1B,CABIN1	10
CREB1	DAXX,NPM1,NASP,ATRX,UBN1,HJURP,DNAJC9,CABIN1,MCM2,ASF1B	10

TF, Transcription factor; Overlapping genes, the genes may be regulated by TF; count, the count of overlapping genes.

Supplementary Table S5. The result of correlation analysis of H3F3B, HIST1H4I and CEPN-A with each H3-H4 histone chaperone

	H3F3B		HIST1H4I		CENPA	
	Coeffecient	P.value	Coeffecient	P.value	Coeffecient	P.value
APLF	0.39796728	9.49E-13	-0.0472722	0.76794256	0.35759481	2.05E-14
ASF1A	0.28862378	5.58E-10	-0.032481	0.93133314	0.32099314	7.52E-13
ASF1B	0.52984958	7.01E-27	0.08267888	0.13632024	0.88950607	8.01E-113
ATRX	0.40320921	1.33E-16	0.05247883	0.18089285	0.29151822	2.35E-09
DAXX	0.54807407	1.04E-28	0.22981967	1.80E-06	0.49156182	9.41E-25
CHAF1A	0.58336338	7.06E-33	0.08001562	0.12481189	0.76145137	4.56E-70
CHAF1B	0.53162171	5.52E-26	0.01471718	0.83984137	0.84856027	7.17E-86
RBBP4	0.53583228	2.08E-26	0.1440999	0.00302156	0.56623542	9.88E-32
DEK	0.5629125	5.62E-31	0.20584278	7.57E-05	0.56960672	3.49E-31
DNAJC9	0.52162629	1.08E-24	0.10797219	0.02604225	0.65405497	3.59E-42
SUPT16H	0.59339443	1.00E-33	0.12792304	0.00589385	0.56810517	2.30E-34
SSRP1	0.56626699	6.92E-32	0.15383892	0.00060388	0.66081525	5.05E-47
HIRA	0.21070201	7.22E-06	0.11221579	0.02776582	0.28230762	1.53E-09
CABIN1	0.43133352	1.28E-18	0.02887132	0.3721401	0.46328629	7.12E-23
UBN1	0.47949704	6.45E-22	0.12987333	0.00977644	0.40092894	2.46E-16
HJURP	0.47718432	7.12E-21	0.06168509	0.23252917	0.93467114	8.55E-150
MCM2	0.60413641	4.21E-34	0.09140306	0.04782726	0.84264449	3.12E-91
NASP	0.53154349	6.92E-28	0.14725797	0.00217561	0.72125445	6.41E-63
NPM1	0.24410475	4.96E-07	0.1584829	0.00191686	0.46829587	4.32E-21

Supplementary Table S6. The results of multivariate Cox analysis of stages and H3-H4 histone chaperones in TCGA-LIHC

Variates	HR (95% CI for HR)	P.value	Significance
Stage I	1		
II	1.0707 (0.6403-1.790)	0.79458	ns
III	1.7806 (1.1145-2.845)	0.0158	*
IV	7.6157 (2.2800-25.438)	<0.001	***
ASF1A	1.3925 (1.0955-1.778)	0.02402	*
HJURP	1.3174 (1.1488-1.569)	0.01518	*
NASP	0.9962 (0.7244-1.370)	0.98155	ns
NPM1	1.2953 (0.9872-1.700)	0.06194	ns

HR, hazard Ratio; 95% CI, 95% confidence interval; ns, not significant; TCGA-LIHC, The Cancer Genome Atlas Liver Hepatocellular Carcinoma
